# Virtual Terroir and the Premium Coffee Experience

**DOI:** 10.3389/fpsyg.2021.586983

**Published:** 2021-03-18

**Authors:** Francisco Barbosa Escobar, Olivia Petit, Carlos Velasco

**Affiliations:** ^1^Department of Food Science, Faculty of Science and Technology, Aarhus University, Aarhus, Denmark; ^2^Centre for Multisensory Marketing, Department of Marketing, BI Norwegian Business School, Oslo, Norway; ^3^Department of Marketing, Kedge Business School, Marseille, France

**Keywords:** premiumness, consumer experience, virtual reality, atmospheres, coffee, terroir, origin

## Abstract

With its origin-centric value proposition, the specialty coffee industry seeks to educate consumers about the value of the origin of coffee and how the relationship with farmers ensures quality and makes coffee a premium product. While the industry has widely used stories and visual cues to communicate this added value, research studying whether and how these efforts influence consumers' experiences is scarce. Through three experiments, we explored the effect of images that evoke the terroir of coffee on the perception of premiumness. Our results revealed that online images that resembled the broad origin of coffee (i.e., a farm) could influence premiumness expectations of coffee (Experiment 1). Similarly, a virtual reality environment that depicted this broad origin (vs. a control but not a city atmosphere) could enhance the perception of coffee premiumness for non-expert consumers (Experiment 2) and the enjoyment of the experience for coffee professionals (Experiment 3). Importantly, we found that congruence between the coffee and the virtual reality (VR) atmospheres mediated how much non-experts enjoyed the experience (Experiment 2). VR atmospheres also influenced expectations of sweetness and acidity for non-experts (Experiment 2). These findings serve as a steppingstone for further exploration of the effects of congruence between visual cues and product/brand attributes on premiumness expectations and perception, and more generally on consumer experience. From a practical standpoint, this study provides insights into key aspects for the development of immersive virtual product experiences.

## 1. Introduction

In the last decades, the specialty coffee industry has expanded internationally and has experienced a significant rate of growth (Morris, 2017). As of 2018, the global specialty coffee market was valued at more than USD 35 billion in revenue, and it is expected to reach USD 85 billion by 2025 (Globe Newswire, 2019). The key factor that grants specialty coffee the status of a premium, much sought-after product lies in the close relationships that coffee companies develop with farmers through the valorization of the coffee's origin, traceability, and potentially the improvement of the production process (Perez et al., 2017). The combination of these factors results in unique flavor profiles. According to the World Intellectual Property Organization (2017), specialty coffee consumers are willing to pay higher prices for coffee, and in exchange, they want to know the origin of the beans and how they were farmed. Therefore, the specialty coffee industry strives to communicate the origin-centric value of coffee in order to improve premiumness perception and thus, increase the willingness of consumers to pay higher prices for the coffee and experiences around it (Perez et al., 2017).

To show that their coffee is a premium product, companies use different cues to convey information about the origin of the coffee. They create flavor profiles linked to the origin of the coffee, which are accompanied by detailed information and stories about the farmers and the terroir (Sanz-Uribe et al., 2017). Additionally, in their websites and packaging, companies present a plethora of images of the terroir and the people that take part in the production (Yeretzian et al., 2017; Gerard et al., 2019). Nevertheless, the outcome of many of the above-mentioned efforts is unclear. To our knowledge, there is no conclusive evidence that indicates that the images about the coffee origin augment the perception of product premiumness and overall experience around coffee. More generally, research on the effect of congruence between visual cues associated with the place of origin and a given product on specialty coffee premiumness perception and experience is scarce. Therefore, there is an opportunity to both understand how images with specific contents, as well as origin congruence, may influence the specialty coffee experience.

In this study, we investigate this topic and go beyond by using virtual reality (VR) as a tool to analyze how atmospheric cues associated with terroir may influence the premium coffee experience. Today, new technologies like VR can create immersive experiences by detaching people from the physical reality, and thus “transporting” them into a virtual environment (Animesh et al., 2011; Gabisch, 2011). In particular, in Experiment 1, we examined whether online images (e.g., photographs on a website) with different levels of congruence with the broad origin of coffee can influence premiumness expectations in coffee. In Experiment 2, we went beyond 2D images and explored whether VR atmospheres that portray the broad origin of coffee can influence how non-expert consumers experience the coffee. In Experiment 3, we extended our investigation to professionals as they appear to rely less on extrinsic cues (e.g., D'Alessandro and Pecotich, 2013; Lee et al., 2018).

## 2. Theoretical Background

### 2.1. Premiumness and Terroir

While there does not seem to be a clear-cut consensus regarding the definition of premiumness, it has been suggested that superior quality is an indispensable element of premium products (Quelch, 1987; Pombo and Velasco, 2020). The literature also seems to agree that premium products add value to the consumer and demand premium prices (see Vigneron and Johnson, 2004; Miller and Mills, 2012; Ko et al., 2019 for reviews on premiumness and luxury goods). Indeed, Quelch (1987) suggested that premium products command the highest quality distribution channels and premium prices that are linked to the performance of the products (see also Lyons and Wien, 2018; Velasco and Spence, 2019 for a review).

For specialty coffee, high quality is a sine qua non-element that is closely associated with the place it comes from (Jackson, 2013). For food and drinks, the concept of origin is especially important since it can convey cultural meaning and lead to the formation of identities, which adds value beyond its purely utilitarian function (Bech-Larsen et al., 2007). The specialty coffee industry, in particular, aims to leverage its connection with the origin of the coffee and its producers to differentiate itself and gain a competitive advantage against the commodity coffee industry (Perez et al., 2017).

For coffee, the concept of terroir is highly relevant in order to analyze the effects of place of origin. Terroir refers to the terrain where a food product comes from that gives it its unique characteristics (Barham, 2003). Terroir is more than a mere geographical link between product and land. It relates to the idea that products are a unique expression of different environmental and sociocultural characteristics of a specific place (Vaudour, 2002). However, it is worth noting that while the specialty coffee industry strives to capitalize on traceability and the use of terroir, it is a relatively young industry (Sepulveda et al., 2016; Perez et al., 2017). Consequently, several consumers know little about the origin of coffee, or they do not see its relevance.

The use of terroir can serve as an indicator of authenticity (a dimension often included in luxury products) with specific geographic heritage (Kuznesof et al., 1997; Beverland, 2006), and it can inhibit the perception of commoditization (Demossier, 2004). The use of terroir can also influence the perception of quality (Heslop et al., 2010) and consumers' willingness to pay higher prices (Livat and Vaillant, 2006; Schamel, 2006; Spielmann et al., 2014; Moulard et al., 2015). Spielmann et al. (2014) suggested that these effects occur mainly because indications of origin provide information and signal quality and authenticity. Highlighting the value of terroir has the potential to enhance product premiumness expectations and perception (Caniato et al., 2009). Research in the wine industry provides evidence that the use of terroir can increase the perception of premiumness (Spielmann and Babin, 2011; Moulard et al., 2015). A useful body of literature to understand why the use of images of terroir might influence perceptions of premiumness in these products is that of schema congruence.

### 2.2. Enhancing Premiumness Associations Through Congruence With Terroir Images

As Douglas and Hargadon (2000) stated, schemas are a “cognitive framework that determines what we know about the world” (p. 154) acquired through previous experiences. These schemas make it possible to make sense of future unfamiliar experiences since they influence information processing and comprehension. Furthermore, congruence relates to the “extent to which a brand association shares content and meaning with another brand association” (Keller, 1993, p. 7). Keller argued that congruent new information is more easily learned and recalled, which determines the cohesiveness of the product-experience image. According to Lee and Labroo (2004), stimuli congruent with a brand or product reduces uncertainty since they share a common meaning, so the product is recalled more easily, becoming semantically predictive (e.g., an advertisement featuring a thirst-quenching beverage in a sporting event). Throughout this paper, we use the terms congruence and fit interchangeably.

Based on these concepts, consumers' previous knowledge about the origin of coffee represents the schema. We construe that schema congruence arises from the interaction and shared common meaning of place of origin between the schema and stimuli that portrays the terroir of coffee. Congruence may generate a cohesive mental image between the new experience and a schema, resulting in basic positive evaluations in terms of familiarity, acceptability, and liking (Mandler, 1982). Consumers prefer the reduced levels of uncertainty brought by congruence as less uncertainty facilitates the processing of new information (Schwarz, 2004). Consequently, the higher degree of fluency that arises in the case of congruence triggers more favorable product evaluations (Winkielman et al., 2015; see also Tofighi et al., 2020 for a more recent example of congruence on brand perception).

Another concept that can serve to analyze the effect of images of a product's terroir on product evaluation is situational appropriateness. It refers to how well a food or beverage product fits the situation in which it is supposed to be consumed (Giacalone and Jaeger, 2019b). Research on this concept suggests that consumers not only evaluate products based on their preferences but also on the situations in which they were meant to be used. Giacalone and Jaeger (2019a) found that perceived situational appropriateness is an essential criterion of product evaluation that can explain over 70% of product choice variance. There are multiple ways in which brands can relate a product with its corresponding terroir, including names, labels, and visual cues through multiple channels. For example, companies can use brand names, labels, and certifications (e.g., AOC, PDO, PGI, TSG) to indicate the provenance of their products (Leclerc et al., 1994; Aichner, 2014). Considering the strong emphasis of terroir products around experiences, hedonic consumption, and knowledge, Charters et al. (2017) suggested that using images about the product and, importantly, its place of origin (landscape, architecture, history, culture) is critical for brands to heighten consumer experiences and create ties to the products and places. Indeed, Häubl and Elrod (1999) highlighted the positive effect of congruence between brand names and country of production on quality perception. In that sense, congruence between visual cues and a product may facilitate premiumness associations. Given that quality is a crucial dimension of brand premiumness, product-origin congruence might lead to higher premiumness perception. Hence, we expected images that more closely resemble the origin of a product to create a higher perception of premiumness. More formally, we hypothesized as follows:

**H1**: Higher (vs. lower) congruence between a given product and visual cues of terroir will result in a higher perception of premiumness.

In addition to the potential effects of visual cues that relate to the origin of products on the perception of coffee premiumness, terroir may influence intent to purchase. As evidenced in the tourism literature, images about a specific location can directly and indirectly—through trip quality, perceived value, and satisfaction—influence intentions to revisit and recommend destinations (Chen and Tsai, 2007). Such images can also influence seek for knowledge and feelings toward the destination (Hosany et al., 2006). As Willems et al. (2019) found, VR images of destinations can generate consumer engagement and increase intent to purchase. Thus, we expected that higher congruence would influence consumers' willingness to buy a product. More formally, we proposed the hypothesis as follows:

**H2**: Higher (vs. lower) congruence between a given product and visual cues of terroir will increase the intent to purchase the product.

Furthermore, we expected the effects of congruence to influence the overall product experience. Visual cues can trigger mental simulation of experiences (Elder and Krishna, 2012), and the use of terroir can imbue more context to these experiences as it can transport consumers to the place of origin (Vaudour, 2002; Charters et al., 2017). For instance, the use of terroir can generate a strong sense of involvement with a product, which in turn can enhance the overall experience and trigger a desire to “become one” with the place (Beverland and Farrelly, 2010). Evoked experiential contexts like terroir can help consumers develop emotional connections with products (Piqueras-Fiszman and Spence, 2015; Charters et al., 2017; Motoki et al., 2020a). Moreover, the use of terroir can provide consumers with connecting experiences with products, the makers, and the place (Smith Maguire, 2010). Taken together, the potential effects of the use of terroir can increase how much consumers enjoy experiences associated with specific products. Thus, we proposed the hypothesis as follows:

**H3**: Higher (vs. lower) congruence between a given product and visual cues of terroir will increase the enjoyment of the overall experience of the product.

### 2.3. Opportunities in Evoking Terroir Through Immersive Technologies

The concepts of presence and telepresence are critical to understand immersive experiences. Presence relates to the perception or sense of being in an environment, where the surroundings have a direct effect on the senses, and telepresence relates to the experience of presence via a communication medium (Steuer, 1992). Moreover, in his seminal paper, Steuer (1992) defined VR as a “real or simulated environment in which the perceiver experiences telepresence” (p. 75). In other words, VR refers to a mediated experience of presence. VR, compared to desktops and mobile phones, can induce more positive emotions and greater psychological and behavioral engagement in destination-based experiences (Flavián et al., 2020). Therefore, VR can be a useful tool for the specialty coffee industry to communicate the concept of terroir and create unique experiences (see also Mollen and Wilson, 2010).

Given the possibilities VR brings to consumer contexts, there has been a growing number of articles on VR in sensory and consumer science in recent years (Spence et al., 2016; Sinesio et al., 2018; Stelick et al., 2018; Andersen et al., 2019; Hannum et al., 2019; Petit et al., 2019; Picket and Dando, 2019; Chen et al., 2020). VR is becoming an effective tool for companies to develop immersive brand experiences with the potential to transport consumers to specific locations an allow them to “live” stories (see also Rogers, 2018). Creating immersive experiences based on real scenarios revolving around terroir in VR can increase consumer engagement and involvement with products and brands (Zandstra et al., 2020). At a low level, VR experiences can foster consumers' desire to continue engaging in these experiences. At a high level, VR can be a crucial tool for companies to more effectively convey the meaning and personality of their brands and help consumers grasp issues related to their products (Hollebeek et al., 2020). Moreover, companies can use VR to develop consumers' emotional connections with brands and products (Harris et al., 2020). As Chirico and Gaggioli (2019) found, the emotions and sense of presence elicited by immersive 360-degree videos are comparable to those elicited by real-life environments. Given the nature of the product and the focus on the stories about terroir and people, the specialty coffee industry may capitalize on VR to tell stories about coffee terroir and farmers to better convey the value and differentiation they add to a product most people consider a mere commodity. In this paper, we tested the effect of congruence in different means that can be used to exhibit terroir. More specifically, we used online 2D images (Experiment 1) and 360-degree VR atmospheres (Experiments 2 and 3).

## 3. Experiment 1: Coffee-Image Congruence and Expectation of Premiumness in Non-experts

The aim of Experiment 1 was to explore whether variations in visual cues used to describe and promote coffee online could be used to manipulate the fit between these cues and the coffee. We began with online images in a product information communication context as it is a common way the industry uses to promote specialty coffee and is one of the first lines in non-expert consumers' evaluation of the coffee. Given the importance of origin in specialty coffee, the variations in the visual cues were done in terms of broad origin, specific origin, and labels, as well as the images themselves, as they can be used to portray different degrees of closeness to the origin of the coffee presented (Leclerc et al., 1994; Aichner, 2014; Charters et al., 2017). Moreover, using different cues, rather than one, can facilitate premiumness associations (Leclerc et al., 1994; Häubl and Elrod, 1999; Aichner, 2014). We sought to uncover whether these factors independently, or interactively, could drive any effect on the fit and premiumness expectations of coffee. We evaluated this as one could also hypothesize that just denoting the country of origin (and assuming that not all consumers know details about the specificities of the origin) could also impact the fit and premiumness expectations. Moreover, we wanted to uncover whether, despite incongruencies between the origin stated in the coffee description and the image, any potential effect of the factors on fit and expectation of premiumness remained. Additionally, the experiment aimed to analyze the effect of this potential fit on premiumness expectations of coffee in non-experts. This experiment also guided the generation of stimuli for the subsequent studies.

### 3.1. Methods

#### 3.1.1. Participants

A total of 770 individuals (516 female, age range 18–74, *M* = 36.5 years, *SD* = 13.5) participated in the experiment.^1^ Participants were recruited from Prolific Academic. All participants were native English speakers and were based in the UK. In Experiment 1, we focused on non-experts. Individuals who participated in this experiment were remunerated with GBP 1.00. This and all the experiments reported in this manuscript were implemented on Qualtrics and complied with the World Medical Association's Declaration of Helsinki. Before beginning each experiment, participants provided their consent to take part in the experiment.

#### 3.1.2. Apparatus and Materials

The stimuli consisted of descriptions of four different specialty coffees along with a photograph and potentially a label of the photograph. The descriptions and photographs of the coffees were selected from those traded by Nordic approach, a green coffee sourcing company based in Oslo, Norway, in the online green coffee marketplace Cropster Hub. The coffees selected were from Burundi, Kenya, El Salvador, and Honduras. The stimuli were created from the combination of three factors (*broad origin, specific origin*, and *label*), which we expected to yield different levels of fit, and the four coffees. Based on the abovementioned literature on terroir, the factor broad origin could take the value of *farm* or *city*, indicating whether the photograph was from a coffee farm or a city. The factor specific origin could take the value *from origin* or *not from origin*, where the former meant the image matched the origin of the coffee in the description. The factor label could take the value of *label* or *no label*, indicating whether the image was labeled with the information about the specific region and country of the image. The factor broad origin was chosen as it could easily portray something close to the terroir of the coffee (farm) vs. something further away from it (city), and as such, we expected it to have the largest effect.

Information about origin can be presented in multiple ways. In this experiment, we were interested in the terroir images themselves. Note, however, that we explored how cues of origin typically used by the specialty coffee industry (e.g., labels) would influence consumer experiences. Even though non-experts may be unfamiliar with coffee-producing countries, the factor-specific origin allowed us to create levels of complete (in)congruence and to analyze how disruptive the stimuli with labels from origin and not from origin were. It also permitted us to analyze which cues would be the most relevant when signaling premiumness. The factor label was chosen as we wanted to know if just looking at a picture portraying nature was enough to influence expectations or if more information was needed. In this experiment, we were interested in generating different levels of congruence while maintaining relative closeness to the origin. Hence, both the farm and the city images were from coffee-producing countries.

**Figure 1:**
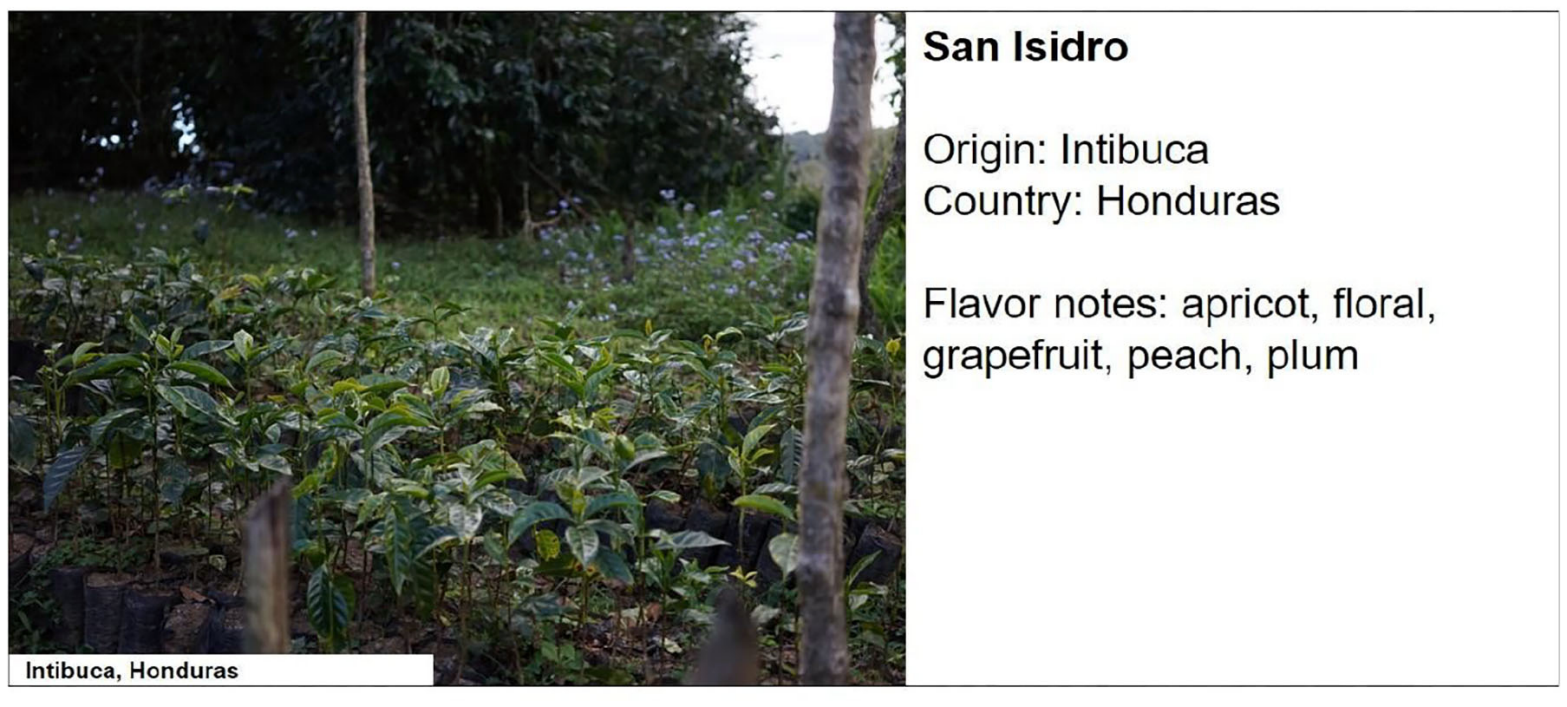
Example of stimuli used in Experiment 1. The factors broad origin, specific origin, and label take the values of farm, from origin, and label, respectively.

All the photographs of the farms were extracted from Cropster Hub, and they were from the specific farms of each of the coffees used in the experiment. The images of cities were from the region where the coffee was produced, and they were taken from Nordic approach's database or free-to-use images. The combination of the three factors and four different coffees resulted in 32 different stimuli. The final stimuli presented to participants consisted of the description of a specific coffee (i.e., name of the farm, specific place of origin, country of origin, the coffee's flavor notes) along with an image placed to the left of the description with or without a label in the lower-left corner. The description was presented in black Arial font, size 18, with a white background. Each stimulus had dimensions of 1362 ×596 pixels and a resolution of 150 dpi (see Figure 1). The complete set of stimuli can be found in OSF at https://osf.io/5vne6/.

We measured premiumness expectations along four dimensions, by adapting Ko et al.'s (2019) luxury dimensions to premium product perception: quality, authenticity, willingness to pay a premium price, and also general premiumness. Participants answered to these dimensions by indicating their level of agreement to a series of statements using five-point Likert scales ranging from 1 (*Strongly disagree*) to 5 (*Strongly agree*). In addition, participants were asked to indicate how well they believed the image fit the coffee through a five-point Likert scale ranging from 1 (*Not well at all*) to 5 (*Extremely well*). See Supplementary Figure 1 for an example of the questionnaire.

To get a sense of the profile of respondents and their relationship with specialty coffee, we included questions regarding their coffee consumption habits and motivations, the factors they considered most important when purchasing coffee, and how familiar they were with specialty coffee and with each of the countries of origin of the coffees presented. See Supplementary Table 1 for a description of participants' profile.

### 3.2. Experimental Design and Procedure

The experiment followed a 2 (broad origin: farm vs. city) ×2 (specific origin: from origin vs. not from origin) ×2 (label: label vs. no label) between-subjects design. Each participant evaluated the four different coffees and was randomly assigned to one of the eight groups. They provided their consent before taking part in the experiment and then proceeded to indicate their age and gender. Afterwards, participants answered the questions regarding their relationship with specialty coffee. Participants later saw each stimulus one at a time and responded to the questions regarding premiumness and the degree of fit between the image and the coffee. The order of the stimuli and premiumness items was randomized. The experiment lasted for approximately 5 min.

### 3.3. Analyses

A mixed analysis of variance (ANOVA) with broad origin, specific origin, and label as between-subjects factors and coffee as within-subjects factor—to control for any difference in perception related to the coffee being evaluated—was conducted to evaluate our dependent variables (fit and premiumness). The measure of effect size for all the ANOVAs was the partial eta squared ($\eta_{p}^{2}$). All the statistical analyses were conducted using R software (R Core Team, 2020). Whenever the interaction terms were significant, Bonferroni-corrected pairwise comparisons were conducted.

Note that premiumness consisted of the average of the four items used to measure it. To check the consistency of the premiumness variable, Cronbach's alpha was computed, yielding a value of 0.75 (95% confidence interval [CI]: 0.74, 0.77), which exceeded the recommended threshold of 0.70 (Nunnally, 1994).

### 3.4. Results and Discussion

#### 3.4.1. Fit Between Visual Cues and Coffee

**Figure 2:**
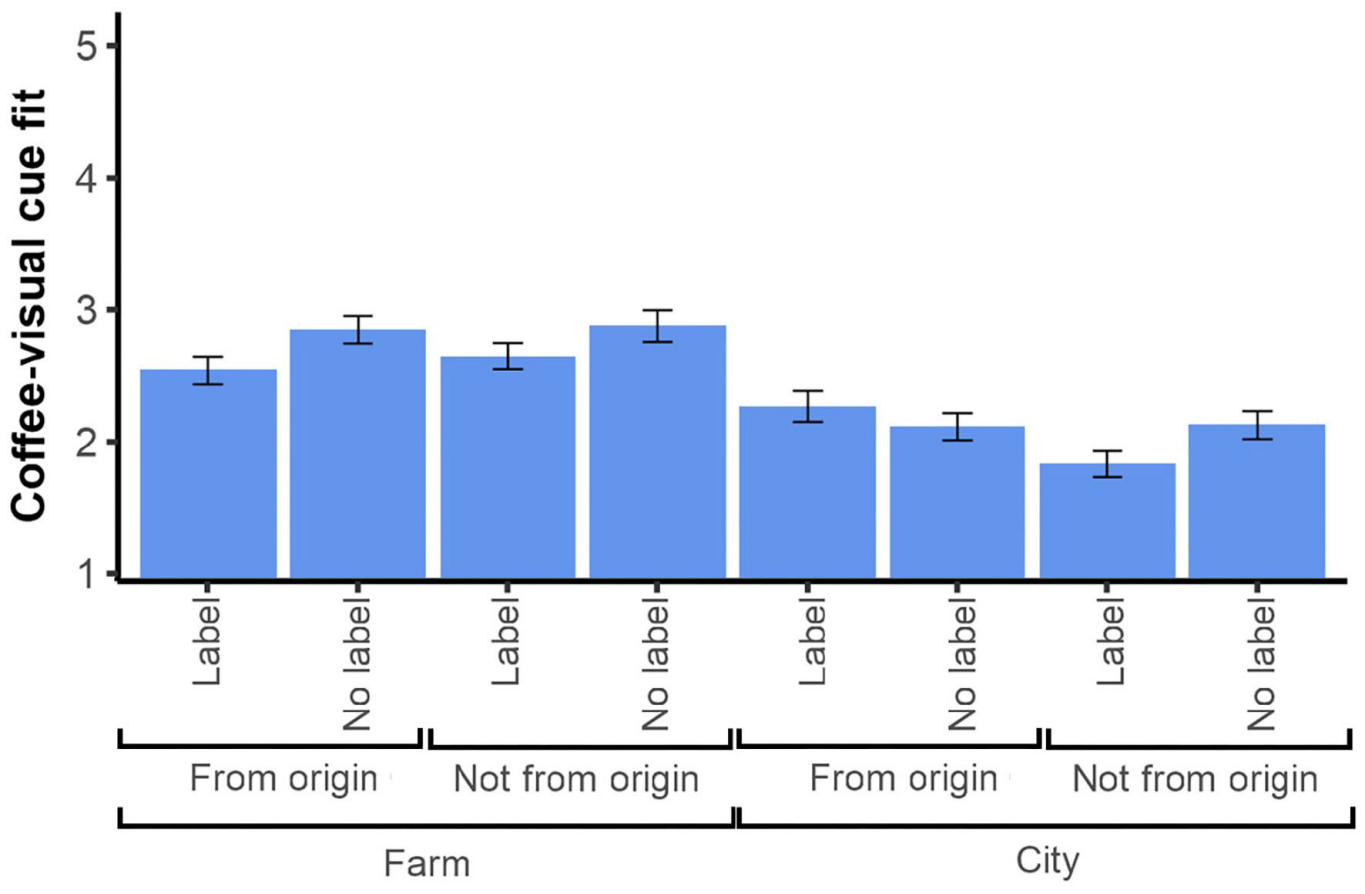
Mean fit ratings between visual cues and coffees in Experiment 1. Ratings of fit on a 1–5 scale. Error bars: 95% CI.

The analysis confirmed the effectiveness of the fit manipulation (Figure 2). The analysis revealed that all main effects and interactions were significant (Table 1A). As for the main effects, farm images (*M* = 2.72, *SD* = 1.09) presented higher fit with the coffee than city images (*M* = 2.08, *SD* = 1.05). Moreover, the images from the specific origin of the coffee (*M* = 2.45, *SD* = 1.11) presented higher fit than those not from the specific origin (*M* = 2.36, *SD* = 1.13). Surprisingly, the stimuli without labels (*M* = 2.49, *SD* = 1.11) presented higher fit ratings than those with labels (*M* = 2.33, *SD* = 1.12). As expected, broad origin was the factor with the largest effect size.

**Table 1:** Analysis of variance (ANOVA) results on fit **(A)** and premiumness **(B)** for Experiment 1.

	**(A) Fit**				**(B) Premiumness**		
**Factor**	***F***	***p***	$\eta_{p}^{2}$		***F***	***p***	$\eta_{p}^{2}$
Coffee	**2.74**	**0.042**	**0.003**		**14.96**	** <0.001**	**0.014**
Broad origin	**280.33**	** <0.001**	**0.085**		**15.18**	** <0.001**	**0.005**
Specific origin	**6.05**	**0.014**	**0.001**		0.14	0.706	<0.001
Label	**21.12**	** <0.001**	**0.007**		0.63	0.426	<0.001
Broad origin × Specific origin	**13.24**	** <0.001**	**0.005**		**6.71**	**0.010**	**0.002**
Broad origin × Label	**6.62**	**0.010**	**0.002**		0.26	0.610	<0.001
Specific origin × Label	**5.14**	**0.023**	**0.002**		3.16	0.076	0.001
Broad origin × Specific origin × Label	**11.96**	** <0.001**	**0.004**		**3.86**	**0.050**	**0.001**

*Bold values indicate the statistically significant effects at p <0.05*.

To further analyze the effects of the significant two-way interactions, pairwise comparisons were conducted. First, when the visual cues were not from the specific origin, farm images (*M* = 2.75, *SD* = 1.10) presented higher fit than city images (*M* = 1.97, *SD* = 1.03; *p* <0.001), and when the cues were from the specific origin, farm images (*M* = 2.69, *SD* = 1.09) also presented higher fit compared to city images (*M* = 2.18, *SD* = 1.07; *p* <0.001). Second, when the images were not labeled, those portraying farms (*M* = 2.86, *SD* = 1.08) presented higher fit than those portraying cities (*M* = 2.12, *SD* = 1.02; *p* <0.001). Similarly, when the images were labeled, those with farms (*M* = 2.59, *SD* = 1.09) presented higher fit than those with cities (*M* = 2.03, *SD* = 1.09; *p* <0.001). Lastly, when the images were labeled, those from the specific origin (*M* = 2.41, *SD* = 1.12) were rated as having higher fit than those not from the specific origin (*M* = 2.25, *SD* = 1.12; *p* = 0.003). However, there was not a significant difference between images from the specific origin and not from the specific origin when they were not labeled (*p* = 0.968).

**Figure 3:**
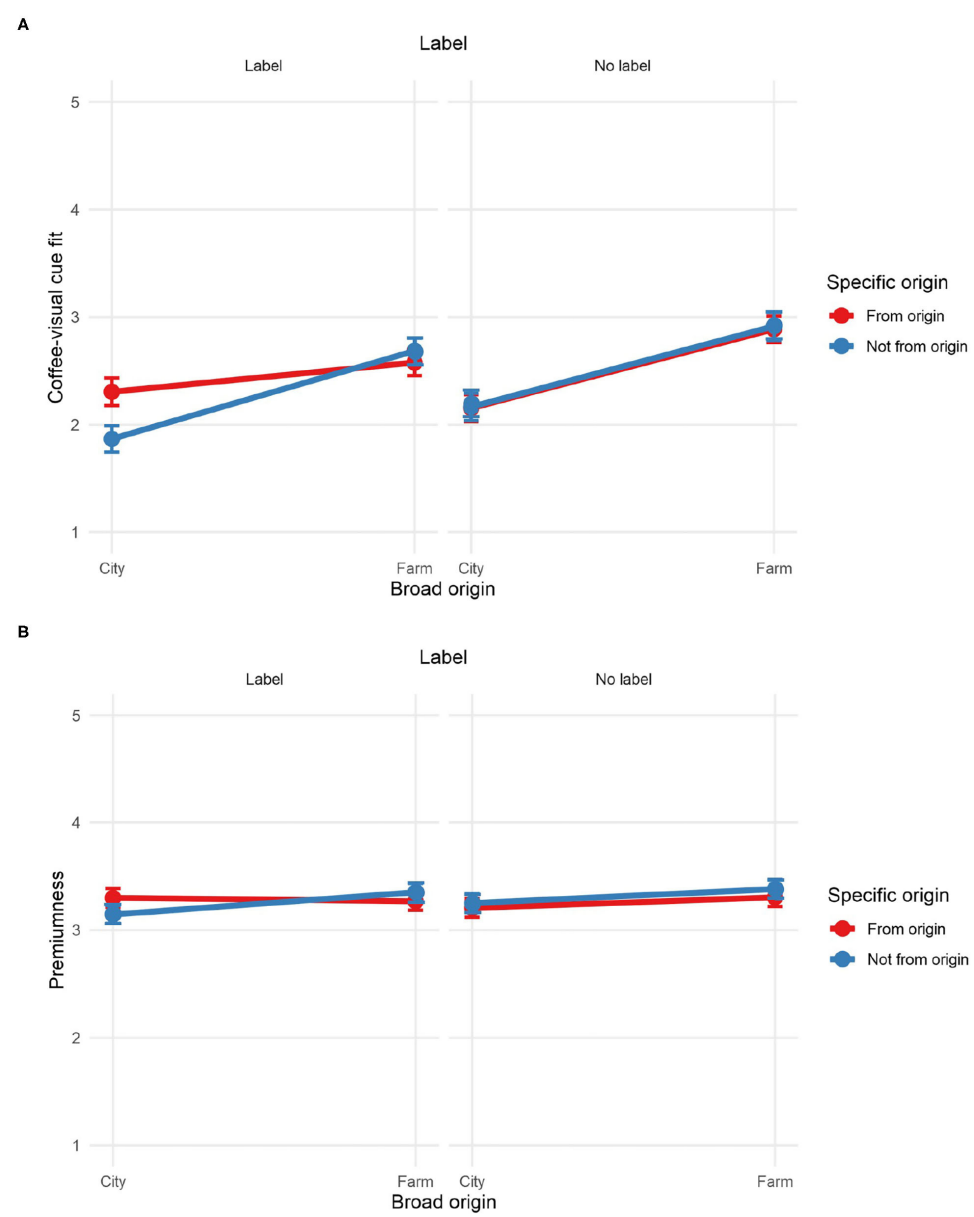
Mean ratings of **(A)** fit and **(B)** premiumness in Experiment 1. Ratings of fit and premiumness on a 1–5 scale. Error bars: 95% CI.

To further investigate the effects of the significant three-way interaction (Figure 3), two separate ANOVAs for the label and no label conditions were conducted. In the former case, the analysis revealed significant main effects of broad origin, *F*_(1, 3069)_ = 110.0, *p* <0.001, $\eta_{p}^{2}$ = 0.035, and specific origin, *F*_(1, 3069)_ = 8.14, *p* = 0.004, $\eta_{p}^{2}$ = 0.003, as well as a significant interaction effect of broad origin and specific origin, *F*_(1, 3069)_ = 26.5, *p* <0.001, $\eta_{p}^{2}$ = 0.009. Subsequent pairwise comparisons of the latter showed that within the labeled images from the specific origin, those that portrayed farms (*M* = 2.54, *SD* = 1.11) had higher fit than those that portrayed cities (*M* = 2.27, *SD* = 1.12; *p* <0.001). Similarly, from the images with labels and not from the specific origin, those that depicted farms (*M* = 2.65, *SD* = 1.07) presented higher fit than those that portrayed cities (*M* = 1.83, *SD* = 1.01; *p* <0.001). Under the no label case, the ANOVA revealed a significant main effect of broad origin, *F*_(1, 3069)_ = 180.0, *p* <0.001, $\eta_{p}^{2}$ = 0.055.

Broad origin appeared to be the most important factor in the manipulation of fit. It is natural to assume that non-experts may not necessarily be able to distinguish when the image (whether from a farm or a city) is not from the specific origin if it does not have a label, which, as the results showed, did not affect the fit. It is interesting to note that the fit ratings of all groups of stimuli were below the midpoint of the scales, likely indicating that participants did not have strong associations between the coffee and the visual cues resembling its origin. One possibility is that participants regarded coffee as an undifferentiated product, and the images provided little specific meaning.

#### 3.4.2. Expectation of Premiumness

The three-way ANOVA (Table 1B) revealed a significant, albeit small, main effect of broad origin (farm vs. city) such that coffees with images portraying farms (*M* = 3.35, *SD* = 0.78) resulted in higher premiumness ratings than those portraying cities (*M* = 3.25, *SD* = 0.68). The analysis also showed a significant two-way interaction between broad origin and specific origin. Pairwise comparisons revealed that when the visual cues were not from the specific origin, farm images (*M* = 3.39, *SD* = 0.81) were rated as more premium than city images (*M* = 3.22, *SD* = 0.66; *p* <0.001). However, there was not a significant difference between farm and city images when they were from the specific origin (*p* = 0.360).

Furthermore, the ANOVA revealed a significant three-way interaction of broad origin, specific origin, and label. To further analyze its effect, two separate ANOVAs for the label and no label conditions were conducted. Under the label condition, the analysis revealed a significant main effect of broad origin, *F*_(1, 3072)_ = 6.54, *p* = 0.011, $\eta_{p}^{2}$ = 0.002, and a significant interaction effect of broad origin and specific origin, *F*_(1, 3072)_ = 10.7, *p* = 0.001, $\eta_{p}^{2}$ = 0.003. Subsequent pairwise comparisons showed that in the case of images with labels and not from the specific origin, those that portrayed farms (*M* = 3.37, *SD* = 0.79) presented higher premiumness than those that portrayed cities (*M* = 3.17, *SD* = 0.69; *p* <0.001). However, there was not a significant difference between farm and city images when they were from the specific origin and were labeled. Under the no label case, the analysis only revealed a significant main effect of broad origin, *F*_(1, 3072)_ = 8.98, *p* = 0.003, $\eta_{p}^{2}$ = 0.003.

The results of Experiment 1 suggested that the fit between visual cues and coffee can be manipulated through indications of the terroir of the coffee, more specifically images varying in terms of the broad origin portrayed (farm or city), whether they are from the specific origin of the coffee or not, and whether they have labels indicating this specific origin. These findings seemed to indicate that visual cues of terroir can influence expectations of the product. These results provided support for hypothesis H1 and served as an initial step for further exploration. Since the broad origin factor (farm vs. city) had the most prominent effect on fit and premiumness expectations in a product information communication setting, in the subsequent experiments we aimed to evaluate whether the broad origin could also influence the coffee experience. For these experiments, we used VR atmospheres as they can provide a more immersive experience of contexts (congruent and incongruent with the terroir) than 2D images. Hence, VR atmospheres of a city and a coffee farm were used as stimuli for subsequent experiments.

## 4. Experiment 2: Coffee-VR Atmosphere Fit, Premiumness Perception, and Sensory Evaluation with Non-experts

The goal of Experiment 2 was to investigate whether different VR atmospheres, varying in their level of fit with specialty coffee, would influence the perception of coffee premiumness. The selection of the visual stimuli was guided by the findings in Experiment 1, which indicated that broad origin (farm vs. city) was the main factor influencing fit and premiumness. Thus, the stimuli for Experiments 2 and 3 consisted of VR atmospheres of a city and a farm. In Experiment 2, we sought to understand the effect of more immersive visual cues close to the terroir of coffee (vs. far from the terroir) on consumers experiences in non-experts. Moreover, in this experiment, we wanted to widen the difference between the farm and the city in terms of their closeness to origin. We also explored whether such VR atmospheres would influence the perception of the sensory characteristics of the coffee. In this experiment, we moved on to actual coffee aroma evaluations, since it is a typical way in which consumers evaluate coffee prior to making purchasing decisions.

### 4.1. Methods

#### 4.1.1. Participants

A total of 143 participants (39 males) took part in the experiment (age range 18–57, *M* = 26.0 years, *SD* = 7.2). The data corresponding to two participants was excluded from the analyses as they indicated in the questionnaire, they did not have a normal smell function. Hence, the final data consisted of observations from 141 participants. Participants were recruited through a behavioral studies platform at BI Norwegian Business School (Oslo, Norway), and they were compensated with NOK 30 for their time.

#### 4.1.2. Apparatus and Materials

**Figure 4:**
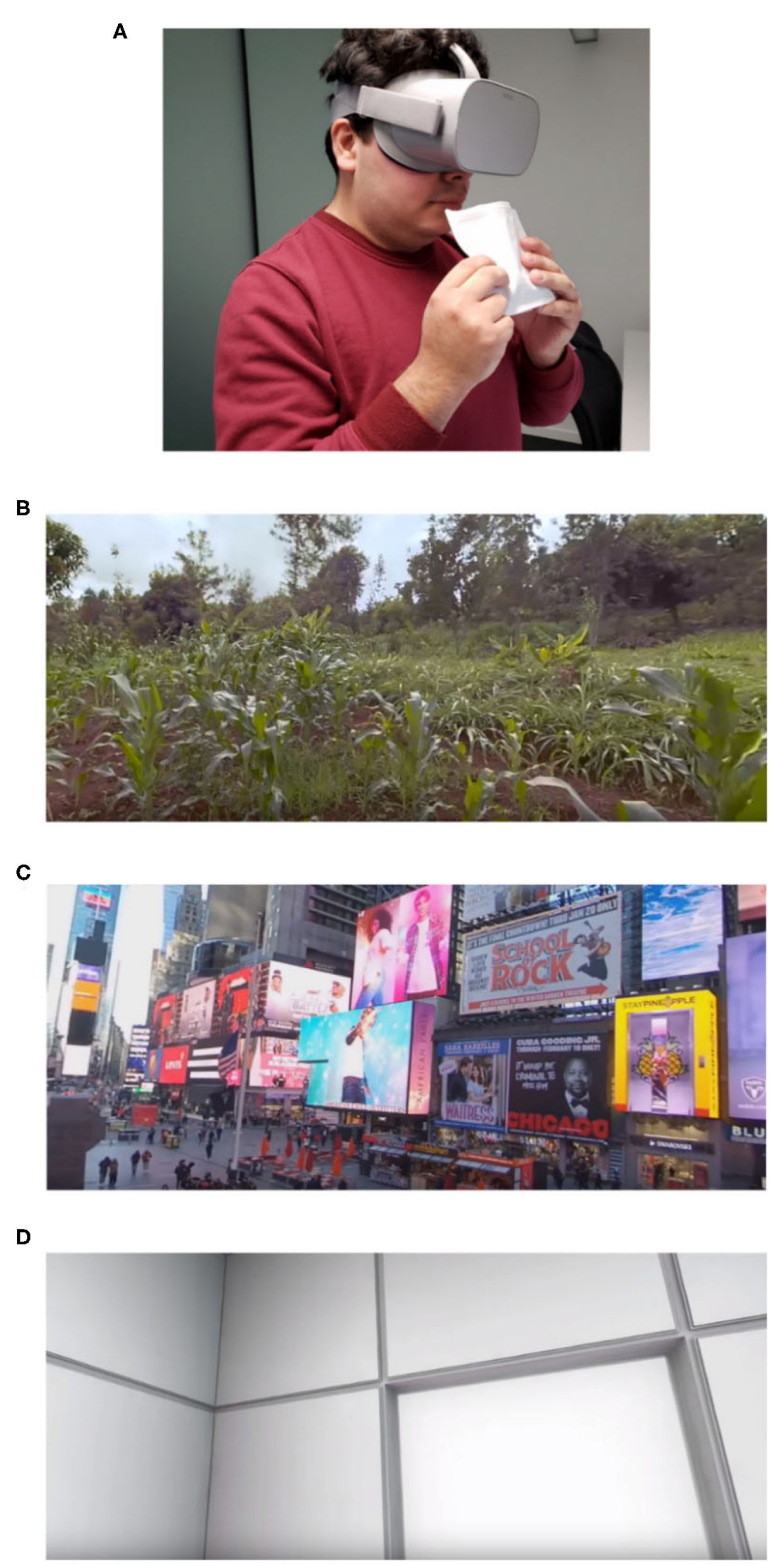
Panel **(A)** presents the instruments used in Experiment 2: Oculus GO virtual reality (VR) headset and sample coffee bag. The remaining panels show partial snapshots of the VR atmospheres used in Experiments 2 and 3: **(B)** farm, **(C)** city, and **(D)** control.

Participants were tasked to smell ground coffee from a small, white, resealable sample bag while exploring a VR atmosphere (Figure 4A). The bags contained approximately 20 g of Kiawamururu coffee from Nyeri, Kenya. The same coffee was used for the three atmospheres. The coffee had flavor notes of raspberries, red apples, and rose hips. To ensure quality and consistency, the coffee was previously ground, flushed with nitrogen (to prevent oxidation), packaged, and sealed into the bags.

The stimuli for Experiment 2 consisted of high definition, 360-degree images—with no sound—of three different atmospheres corresponding to a farm, a city, and a control atmosphere (Figure 4). All the stimuli were presented in an Oculus Go VR headset. The capture of the farm was a coffee plantation in the Kangema Constituency of Kenya.^2^ The image of the city was a capture of Times Square (New York City, United States)^3^. This atmosphere was chosen as we wanted to increase the level of incongruence between the atmosphere from the terroir vs. not from the terroir (farm vs. city). Finally, the control atmosphere consisted of a white room^4^. This atmosphere was chosen as a control as it is far from coffee contexts and relatively neutral. It is worth noting that the farm and the city atmosphere were real-life captures, while the control atmosphere was a computer-generated environment. Participants did not receive any prior information regarding any of the atmospheres (i.e., they were not told that the farm atmosphere was a coffee farm or that it was in Kenya).

In Experiment 2, we measured premiumness and fit as in Experiment 1, though here, participants were asked about the actual coffee aroma. Participants were asked to evaluate eight of the 10 sensory characteristics of the coffee, following the SCAA Arabica cupping form (Lingle and Menon, 2017), using slider scales from 0 to 100. The specific elements evaluated were aroma, flavor, acidity, sweetness, balance, and overall. Aftertaste and body were excluded from this experiment as they can only be evaluated by tasting the coffee. The experiment explored these characteristics as they are the ones that the specialty coffee professionals use when evaluating coffee (Lingle and Menon, 2017). We also asked participants to rate their willingness to purchase the coffee and their enjoyment of the overall experience using a slider scale from 0 to 100.

We included different control questions. Participants were asked to indicate the number of years they had lived in the city and the countryside and how familiar they were with specialty coffee with a five-point Likert scale ranging from 1 (*Not familiar at all*) to 5 (*Extremely familiar*).

Additionally, we included different control variables specific to VR. To measure the sense of presence in the VR environment, participants were asked to evaluate a series of statements on a five-point Likert scale ranging from 1 (*Strongly disagree*) to 5 (*Strongly agree*). More specifically, we used the five items of the Physical Presence sub-dimension of Makransky et al.'s (2017) scale. These five items measured physical realism, lack of awareness of physical mediation, consistency with real-world experiences, sense of being in the virtual environment, and captivation by the virtual environment.

### 4.2. Experimental Design and Procedure

The experiment took place in the school's behavioral laboratory. First, participants provided their consent to take part in the experiment and later proceeded to indicate their age and gender and answer the control questions. Then, they were asked to stand up, were introduced to the Oculus GO VR headset, and were later asked to put the headset in on mode. Participants found themselves in the corresponding VR environment and after a few seconds to get familiar with the setting, participants received the bag of coffee. They were instructed to open the bag and smell the coffee as many times as they wished while they explored the atmosphere and assessed the coffee aroma. Note that participants were not able to see their hands or the coffee bag in the VR atmosphere. Once they finished, they removed the headset and returned the sample bag. Next they proceeded to answer the questions related to their premiumness perception, sensory characteristics, and intent to purchase the coffee, and their experience of the VR environment, using a desktop computer. The experiment lasted for approximately 10 min.

### 4.3. Analyses

The different dependent variables used in this experiment were analyzed by means of one-way ANOVAs. Tukey-corrected pairwise comparisons were conducted when significant effects were observed. Additionally, we conducted a serial mediation analysis since we were interested in the holistic experience and aimed to understand its underlying mechanism. We aimed to uncover whether the potential impact of the atmospheres on the enjoyment of the experience worked directly on this measure or indirectly through other aspects related to the experience. The analyses were conducted using R software (R Core Team, 2020), except the mediation analyses, which were performed with the PROCESS macro for SPSS (Hayes, 2012). The measure of premiumness resulted in a Cronbach's alpha of 0.84 (95% CI: 0.80, 0.89). The sense of presence measure consisted of the average of the five items of the physical realism sub-dimension in Makransky et al. (2017) scale, with a Cronbach's alpha of 0.82 (95% CI: 0.77, 0.86).

### 4.4. Results and Discussion

The summary statistics of all dependent variables are presented in Table 2.

**Table 2:** Descriptive statistics for Experiment 2.

	**City**		**Control**		**Farm**		**Overall**	
	**(*****n*** **= 48)**		**(*****n*** **= 47)**		**(*****n*** **= 48)**		**(*****n*** **= 143)**	
	***M***	***SD***	***M***	***SD***	***M***	***SD***	***M***	***SD***
**Premiumness**								
Quality	3.65^*ab*^	0.79	3.40^*a*^	0.97	3.94^*b*^	0.81	3.66	0.88
Authenticity	3.85^*a*^	0.90	3.57^*a*^	0.99	4.00^*a*^	1.05	3.81	0.99
Premiumness	3.44^*ab*^	0.92	3.11^*a*^	1.03	3.63^*b*^	0.98	3.39	0.99
Willingness to pay a premium	3.06^*a*^	0.93	3.04^*a*^	0.98	3.40^*a*^	0.96	3.17	0.96
Premiumness index	3.50^*ab*^	0.70	3.28^*a*^	0.84	3.74^*b*^	0.78	3.51	0.79
Liking	4.15^*a*^	1.03	4.17^*a*^	1.03	4.38^*a*^	1.00	4.23	1.02
Enjoyment of the experience	81.02^*a*^	15.87	77.87^*a*^	19.23	85.56^*a*^	14.76	81.51	16.89
Intent to purchase	63.54^*a*^	25.35	62.70^*a*^	27.73	69.10^*a*^	26.05	65.13	26.35
**VR environment**								
Coffee-VR atmosphere fit	3.35^*b*^	1.14	2.26^*a*^	1.26	3.60^*b*^	1.05	3.08	1.28
Physical realism	3.40^*b*^	1.16	2.66^*a*^	1.17	3.81^*b*^	1.10	3.29	1.23
Lack of awareness	3.46^*b*^	0.97	2.94^*a*^	1.17	3.29^*ab*^	0.97	3.23	1.05
of physical mediation								
Consistency with experience	3.25^*b*^	1.12	2.64^*a*^	1.21	3.48^*b*^	1.05	3.13	1.17
in the real world								
Sense of being in the	3.75^*b*^	1.12	3.00^*a*^	1.34	3.96^*b*^	1.13	3.57	1.26
virtual environment								
Captivation by the	3.38^*b*^	1.08	2.66^*a*^	1.31	3.48^*b*^	1.13	3.17	1.22
virtual environment								
**Sensory evaluation**								
Aroma	75.17^*a*^	16.97	73.53^*a*^	18.70	79.56^*b*^	20.22	76.10	18.72
Flavor	70.58^*a*^	17.68	71.28^*a*^	17.49	73.77^*b*^	20.29	71.88	18.46
Acidity	51.10^*ab*^	21.10	43.28^*a*^	21.58	54.42^*b*^	22.50	49.64	22.09
Sweetness	44.40^*a*^	22.23	56.23^*b*^	22.18	53.06^*b*^	20.49	51.20	22.07
Balance	65.21^*a*^	18.73	63.49^*a*^	17.91	68.79^*b*^	16.61	65.85	17.78
Overall	73.10^*a*^	16.28	72.15^*a*^	15.21	77.48^*b*^	16.92	74.26	16.21

*Values within rows that do not share the same superscript letter are significantly different as per the Tukey-corrected pairwise comparisons (at p <0.05)*.

#### 4.4.1. Control Variables: Familiarity With the Coffee and Sense of Presence

**Figure 5:**
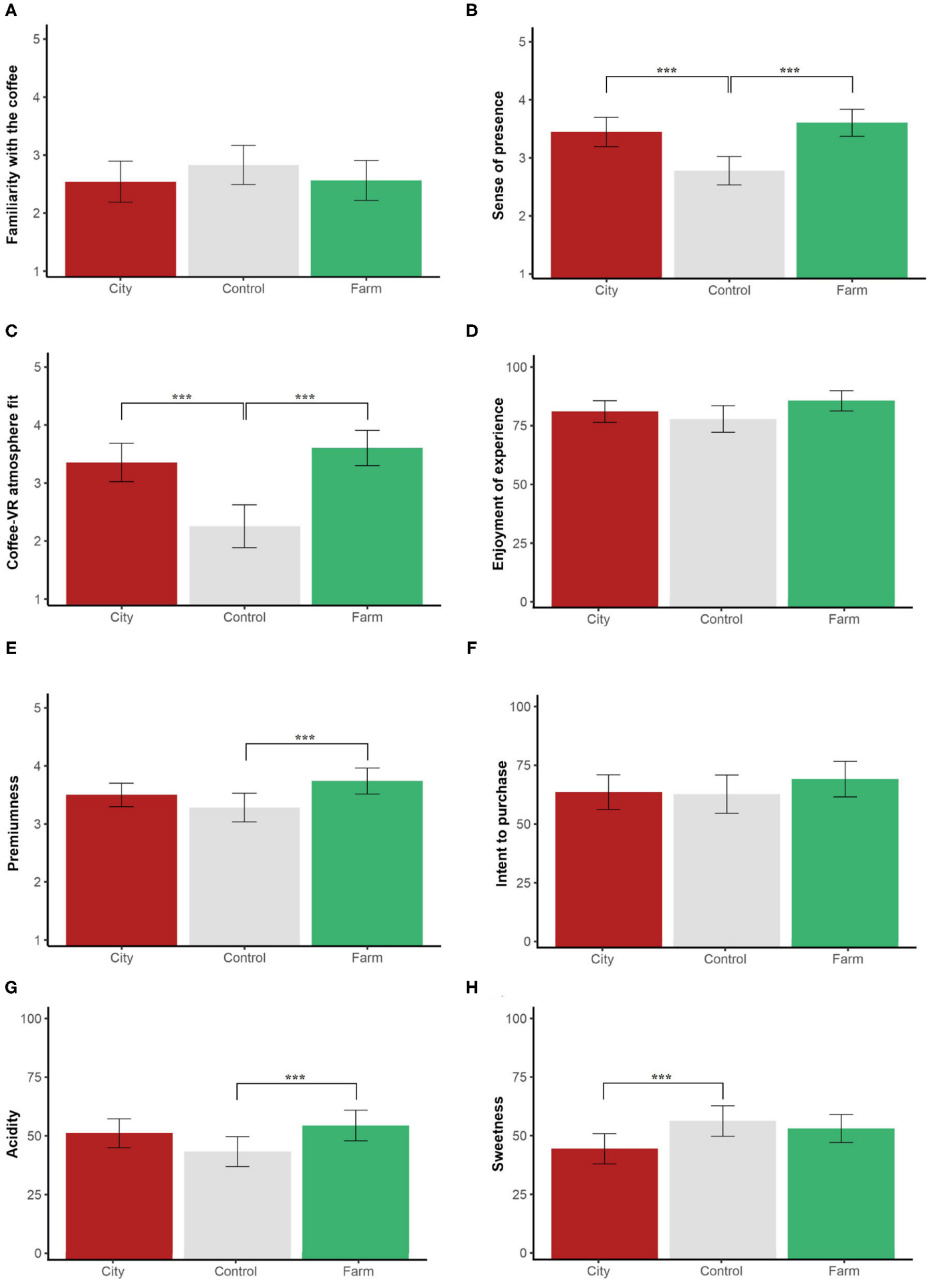
Mean ratings of **(A)** familiarity with the coffee, **(B)** sense of presence, **(C)** coffee-virtual reality (VR) atmosphere fit, **(D)** enjoyment of the experience, **(E)** premiumness, **(F)** intent to purchase, **(G)** acidity, and **(H)** sweetness for Experiment 2. Ratings of **(A–C,E)** on a 1–5 scale. Error bars: 95% CI. Significantly different comparisons: ***p <0.01.

The analysis did not reveal any significant effect of the VR atmospheres on the familiarity with the coffee (Table 3, Figure 5A). However, the analysis revealed a significant effect of the atmospheres on the sense of presence in the virtual environment (Table 3). Pairwise comparisons showed significant differences between the farm and control atmospheres (*p* <0.001) and between the city and control atmospheres (*p* <0.001) (Figure 5B). There was not a significant difference between the farm and the city atmospheres (*p* = 0.625). These results were expected as the city and farm atmospheres were more vivid, with more elements and colors than the control atmosphere, which consisted of a small, white room.

**Table 3:** Analysis of variance (ANOVA) results for Experiment 2.

	***F***	***p***	**$\eta_{p}^{2}$**
Familiarity with the coffee	0.87	0.421	0.012
Sense of presence	**12.95**	** <0.001**	**0.156**
Coffee-VR atmosphere fit	**18.41**	** <0.001**	**0.208**
Premiumness	**4.15**	**0.018**	**0.056**
Intent to purchase	0.83	0.438	0.012
Enjoyment of experience	2.55	0.082	0.035
Aroma	1.33	0.268	0.019
Flavor	0.39	0.677	0.006
Acidity	**3.28**	**0.041**	**0.045**
Sweetness	**3.82**	**0.024**	**0.052**
Balance	1.10	0.335	0.016
Overall	1.48	0.232	0.021

*Bold values indicate the statistically significant effects at p <0.05*.

#### 4.4.2. Coffee-VR Atmosphere Fit

The analysis revealed a significant effect of the atmospheres on the fit between the VR environment and the coffee (Table 3). Pairwise comparisons revealed significant differences between the farm and the control atmospheres (*p* <0.001), and between the city and the control (*p* <0.001) atmospheres. The difference in fit between the farm and city atmospheres was not statistically significant (*p* = 0.538) (Figure 5C). Contrary to our expectations, both the farm and the city atmosphere seemed to be a relatively congruent with the coffee experience compared to the control. Participants may not have found rich meaning in the farm atmosphere perhaps because they were just as equally suitable for the coffee or potentially because they might not have been aware it was a coffee farm. Non-experts may not be familiar with coffee farms, and their expectations of how they look like might differ greatly from reality.

#### 4.4.3. Enjoyment of the Experience, Premiumness Perception, and Intent to Purchase

The ANOVA did not reveal a significant effect of atmospheres on the enjoyment of the experience (Figure 5D), failing to provide support to H3. However, a significant effect of the atmospheres on premiumness was observed (Table 3). Pairwise comparisons revealed a significant difference in premiumness perception between the farm atmosphere and the control (*p* = 0.013) but not between the farm and the city atmospheres (*p* = 0.287) (Figure 5E). Contrary to our expectations, these results only partially supported H1. The analysis did not reveal any significant effect of the atmospheres on intent to purchase (Table 3, Figure 5F), failing to provide support to H2.

Considering that the premiumness index involved a series of scales inspired by Ko et al. (2019), additional ANOVAs were conducted to assess the effect of the atmospheres on the individual components of premiumness and liking. The analyses revealed significant effects only on quality and premiumness, but not authenticity, willingness to pay a premium, or liking (see Supplementary Table 2). Significant differences were found between the farm and the control atmospheres in terms of the quality item (*p* = 0.029), as well as between the farm and the control atmosphere in the premiumness item (*p* = 0.008). It is possible that, despite perceiving the coffee as of higher quality and more premium under the farm atmosphere, participants still regarded coffee as an undifferentiated and ubiquitous product or that they are not greatly involved with the product category, resulting in no significant effects in value-related items (willingness to pay a premium and liking).

Furthermore, to look at the effect of the atmospheres and fit on the intent to purchase the product, we conducted a serial mediation analysis (Model 6 of Hayes, 2012; 10,000 bootstrap samples) with VR atmosphere coded as the independent variable, VR atmosphere fit, enjoyment of the experience, and premiumness as mediators; and intent to purchase as the key outcome variable (VR atmosphere → VR atmosphere fit → enjoyment of the experience → premiumness → intent to purchase). Those participants in the VR city atmosphere (vs. control, β = 1.07, *t* = 4.57, *p* <0.001) and those in the VR farm atmosphere (vs. control, β = 1.32, *t* = 5.64, *p* <0.001) reported significantly higher fit between the VR atmosphere and the coffee. Enjoyment of the experience was then regressed on VR atmosphere fit with a significant direct effect (β = 3.96, *t* = 3.39, *p* <0.001). The effects of city (*p* = 0.66) and farm (*p* = 0.58) atmospheres were no longer significant, suggesting a full mediation. Moreover, we found significant direct effects of enjoyment of the experience on premiumness (β = 0.2, *t* = 6.25, *p* <0.001). VR atmosphere fit also had a significant direct effect on premiumness (β = 0.16, *t* = 3.21, *p* = 0.002). The effect of premiumness (β = 8.93, *t* = 3.61, *p* <0.001), as well as the effect of the enjoyment of the experience (β = 0.76, *t* = 6.77, *p* <0.001), on intent to purchase were significant. The direct effects of VR atmosphere fit on intent to purchase was not significant (*p* = 0.07).

Finally, there were significant indirect effects of VR city atmosphere (vs. control, indirect effect = 0.80, 95% CI [0.17; 1.75] excluded zero) and VR farm atmosphere (vs. control, indirect effect = 0.98, 95% CI [0.20; 2.20] excluded zero) on intent to purchase through VR atmosphere fit, enjoyment of the experience, and premiumness, as mediators. Specifically, we found that the city and the farm atmospheres—as opposed to the control atmosphere—created higher VR atmosphere fit, which improved the enjoyment of the experience, subsequently increasing premiumness, and thereby positively affecting intent to purchase, giving partial support to H1, H2, and H3.

#### 4.4.4. Sensory Evaluation

In terms of the perceived sensory characteristics of the coffee, the analysis revealed a significant effect of atmosphere on acidity and sweetness (Table 3, Figures 5G,H). Participants rated the coffee as more acidic under the farm atmosphere compared to the control (*p* = 0.036). Participants found the coffee sweeter under the control vs. the city atmosphere (*p* = 0.023).

Overall, the results of Experiment 2 showed that the farm and city atmospheres elicited a higher sense of presence compared to the control. Contrary to our expectations, both the city and the farm appeared to be contexts with relatively high congruence with the coffee experience. In other words, participants seemed to associate both environments with coffee. Furthermore, participants rated the coffee as more premium under the farm atmosphere compared to the control but not the city, which showed partial support to H1. The atmospheres indirectly influenced the intent to purchase and the enjoyment of the experience, through VR atmosphere fit and premiumness perception, giving partial support to H2 and H3, respectively. The results showed that atmospheric cues can affect the sensory perception of coffee, in terms of sweetness and acidity. In the next experiment, we sought to explore whether the VR atmospheres could also influence the coffee experience in coffee professionals.

## 5. Experiment 3: Coffee-VR Atmosphere Fit, Premiumness Perception, and Sensory Evaluation With Specialty Coffee Professionals

Specialty coffee has experienced a significant growth in interest that has resulted in a rising number of professionals in the field (Lingle and Menon, 2017). Specialty coffee professionals might evaluate products differently (cf. Spence and Carvalho, 2020). For this experiment,we elaborated our design based on previous research on beer and wine. The former suggests that the knowledge experts develop seems to enhance their recognition memory of beers, though this training does not seem to influence their perceptual abilities (Van Doorn et al., 2020). However, the wine literature suggests that experts tend to rely less on extrinsic cues when evaluating wines (D'Alessandro and Pecotich, 2013; Lee et al., 2018). Due to the specific characteristics that make specialty coffee professionals, we decided to conduct an experiment similar to Experiment 2 only on this population, which would allow us to better understand non-expert consumers by comparing the results of both studies. Experiment 3 explores the effect of the different VR atmospheres on the premiumness perception and sensory evaluation of coffee by industry professionals. As explained in the theoretical background, non-experts form expectations differently compared to professionals. Thus, in this experiment, we moved on to taste with professionals as it is how they evaluate and test coffee.

### 5.1. Methods

#### 5.1.1. Participants

A total of 34 individuals (21 males) participated in the experiment (age range 19–57, *M* = 36.2 years, *SD* = 9.2). Participants were recruited from Nordic approach, different coffee roasteries, and other companies in the specialty coffee industry. The pool of participants consisted of professionals working in the specialty coffee industry with expertise in specialty coffee quality and roasting (including certified cuppers and professional roasters) or at the minimum specialized knowledge and experience in cupping, coffee grades, and roasting.

Although other studies in consumer science involving participants with specialized knowledge have used similar sample sizes (Sauvageot et al., 2006; Valentin et al., 2007; Worch et al., 2010; D'Alessandro and Pecotich, 2013), we acknowledge this as a limitation at the outset. Despite this limitation, though, we decided to present this experiment as it provides relevant insights and a steppingstone for future studies.

#### 5.1.2. Apparatus and Materials

In this experiment, participants were tasked to taste a sample of brewed coffee while exploring a VR atmosphere. Participants tasted approximately 100 mL of AA Muthuaini coffee from Nyeri, Kenya, at approximately 60°C from a standard clear glass. The brewed coffee was kept in a professional-grade thermal carafe and was poured into the glass immediately before each participant was going to taste it. Multiple batches of coffee were brewed during the duration of the experiment in order to ensure temperature consistency. The coffee had flavor notes of black currant, red currant, and lime, and it had a cupping score of 87. The cupping score is a measure of the quality of the coffee and ranges from 0 to 100. The score is obtained in standardized coffee tastings by professionals under controlled circumstances. A score above 85 indicates specialty coffees. The VR atmospheres were the same as those used in Experiment 2. However, to increase the generalizability of the analysis, the 360-degree images for the farm and the city conditions were extracted at slightly different points in time in the videos (at seconds 48 and 32, respectively).

We measured premiumness as in Experiments 1 and 2. Regarding the sensory evaluation, participants evaluated the 10 sensory characteristics from the SCAA Arabica cupping form. Aftertaste and body were added here since they can be evaluated by tasting the coffee. Participants also evaluated how much they enjoyed the drinking experience and how likely they were to purchase the coffee. Finally, they also rated their sense of presence in the atmosphere as in Experiment 2.

### 5.2. Experimental Design and Procedure

**Figure 6:**
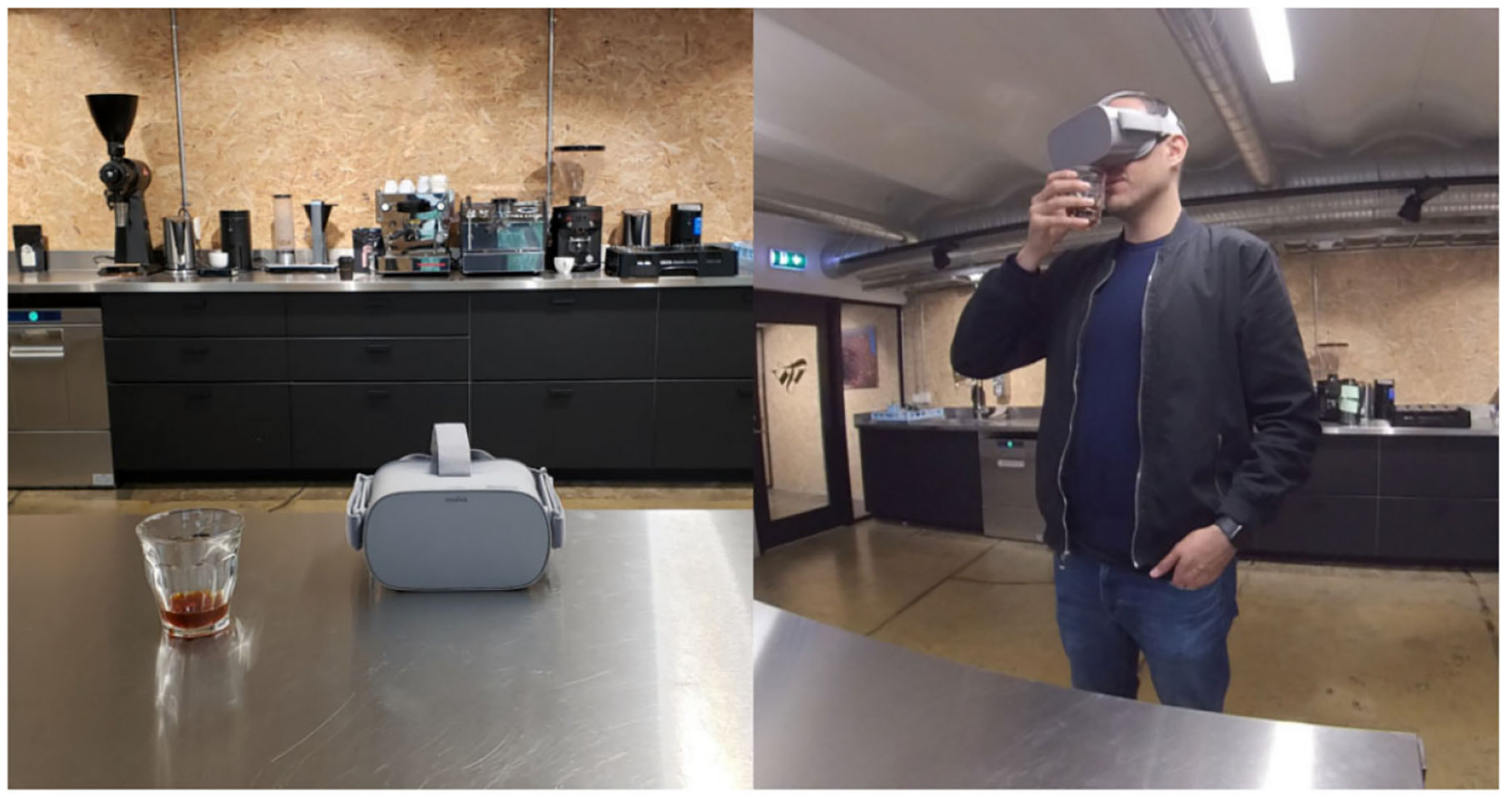
Instruments used in Experiment 3: Oculus GO virtual reality (VR) headset and brewed coffee.

The experiment took place in Nordic Approach's office in Oslo, Norway. The procedure was as in Experiment 2, except that in this case, participants were given a glass with brewed coffee to taste instead of ground beans to smell. Similar to Experiment 2, participants did not see their hands or the cup of coffee in the VR environment. After they tasted the coffee and explored the VR atmosphere, they removed the VR headset and proceeded to complete the questionnaire in their smartphones (Figure 6).

### 5.3. Analyses

The analyses performed were the same as in Experiment 2. The measure of premiumness resulted in a Cronbach's alpha of 0.80 (95% CI: 0.69, 0.91), and the sense of presence resulted in a Cronbach's alpha of 0.74 (95% CI: 0.60, 0.88).

### 5.4. Results and Discussion

Descriptive statistics showed small differences in the various components of premiumness, sensory characteristics, and sense of presence across the different atmospheres (Table 4).

**Table 4:** Descriptive statistics for Experiment 3.

	**City**		**Control**		**Farm**		**Overall**	
	**(*****n*** **= 11)**		**(*****n*** **= 10)**		**(*****n*** **= 13)**		**(*****n*** **= 34)**	
	***M***	***SD***	***M***	***SD***	***M***	***SD***	***M***	***SD***
**Premiumness**								
Quality	4.18^*a*^	0.87	4.40^*a*^	0.52	4.15^*a*^	0.90	4.24	0.78
Authenticity	3.64^*a*^	0.92	4.00^*a*^	0.82	4.31^*a*^	0.95	4.00	0.92
Premiumness	4.18^*a*^	0.60	4.60^*a*^	0.70	4.15^*a*^	1.07	4.29	0.84
Willingness to pay a premium	3.82^*a*^	1.33	3.60^*a*^	0.97	4.23^*a*^	0.73	3.91	1.03
Premiumness index	3.95^*a*^	0.84	4.15^*a*^	0.47	4.21^*a*^	0.78	4.11	0.71
Liking	4.18^*a*^	0.75	4.60^*a*^	0.52	4.23^*a*^	1.01	4.32	0.81
Enjoyment of the experience	69.91^*ab*^	15.10	56.70^*a*^	25.44	79.92^*b*^	17.93	69.85	21.33
Intent to purchase	70.45^*a*^	17.56	68.10^*a*^	11.35	73.69^*a*^	22.78	71.00	17.97
**VR environment**								
Coffee-VR atmosphere fit	2.73^*ab*^	1.19	1.70^*a*^	1.25	3.46^*b*^	0.97	2.71	1.31
Physical realism	3.82^*a*^	0.98	2.70^*a*^	1.25	3.46^*a*^	1.20	3.35	1.20
Lack of awareness	3.18^*a*^	1.25	2.90^*a*^	0.99	2.38^*a*^	0.96	2.79	1.09
of physical mediation								
Consistency with experience	3.18^*a*^	1.25	2.80^*a*^	1.32	2.77^*a*^	1.01	2.91	1.16
in the real world								
Sense of being in the	3.73^*a*^	1.01	3.30^*a*^	1.34	3.46^*a*^	0.88	3.50	1.05
virtual environment								
Captivation by the	3.36^*a*^	1.43	2.20^*a*^	1.23	2.77^*a*^	1.01	2.79	1.27
virtual environment								
**Sensory evaluation**								
Aroma	75.82^*a*^	8.27	68.10^*a*^	14.69	74.23^*a*^	19.84	72.94	15.27
Flavor	75.64^*a*^	11.89	71.70^*a*^	14.35	77.85^*a*^	14.82	75.32	13.62
Aftertaste	68.18^*a*^	23.91	70.30^*a*^	16.45	75.77^*a*^	17.24	71.71	19.14
Acidity	77.82^*a*^	14.62	70.10^*a*^	12.95	81.00^*a*^	10.80	76.76	13.18
Sweetness	72.45^*a*^	20.68	72.10^*a*^	19.08	76.92^*a*^	14.28	74.06	17.56
Body	71.45^*a*^	19.06	70.70^*a*^	14.27	72.31^*a*^	19.51	71.56	17.45
Balance	74.18^*a*^	9.84	72.90^*a*^	10.72	74.54^*a*^	18.16	73.94	13.46
Overall	74.36^*a*^	14.83	75.70^*a*^	10.95	79.31^*a*^	14.27	76.65	13.35

*Values within rows that do not share the same superscript letter are significantly different as per the Tukey-corrected pairwise comparisons (at p <0.05)*.

#### 5.4.1. Control Variables: Familiarity With the Coffee and Sense of Presence

**Figure 7:**
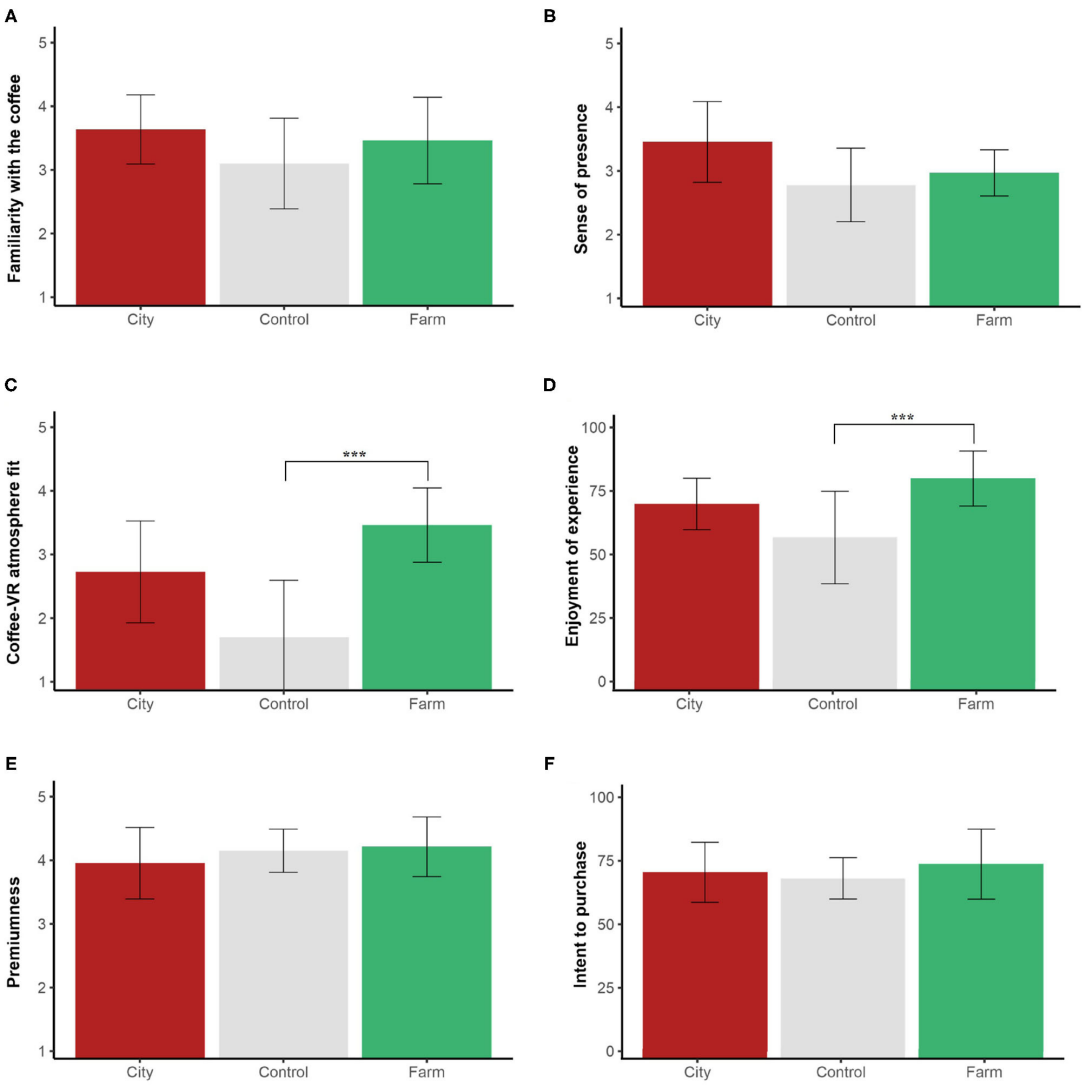
Mean evaluations of **(A)** familiarity with the coffee, **(B)** sense of presence, **(C)** coffee-VR atmosphere fit, **(D)** enjoyment of the experience, **(E)** premiumness, and **(F)** intent to purchase for Experiment 3. Ratings of **(A–C,E)** on a 1–5 scale. Error bars: 95% CI. Significantly different comparisons: ***p <0.01.

Similar to Experiment 2, the analysis did not reveal any significant effect of the VR atmospheres on the familiarity with the coffee (Table 5, Figure 7A). However, unlike non-experts, there were no significant differences among the atmospheres in terms of sense of presence for coffee professionals (Table 5, Figure 7B).

**Table 5:** Analysis of variance (ANOVA) results for Experiment 3.

	***F***	***p***	**$\eta_{p}^{2}$**
Familiarity with the coffee	0.79	0.464	0.048
Sense of presence	2.11	0.138	0.120
Coffee-VR atmosphere fit	**6.88**	**0.003**	**0.308**
Premiumness	0.40	0.676	0.025
Intent to purchase	0.27	0.766	0.017
Enjoyment of experience	**3.95**	**0.030**	**0.203**
Aroma	0.73	0.489	0.045
Flavor	0.56	0.574	0.035
Acidity	2.12	0.137	0.120
Sweetness	0.27	0.766	0.017
Balance	0.04	0.959	0.003
Aftertaste	0.49	0.617	0.031
Body	0.02	0.977	0.001
Overall	0.43	0.655	0.027

*Bold values indicate the statistically significant effects at p <0.05*.

#### 5.4.2. Coffee-VR Atmosphere Fit

The analysis revealed a significant effect of the atmospheres on the fit between the coffee and the VR atmospheres (Table 5). Pairwise comparisons showed a significant difference in fit between the farm atmosphere and the control (*p* = 0.002). However, there was not a statistically significant difference between the farm and city atmospheres (*p* = 0.266) (Figure 7C).

#### 5.4.3. Enjoyment of the Experience, Premiumness Perception, and Intent to Purchase

The ANOVA revealed a significant effect of atmosphere on the enjoyment of the experience (Table 5). Pairwise comparisons showed a significant difference between the farm and the control atmospheres (*p* = 0.023) (Figure 7D), partially supporting H3. However, the analysis did not reveal any significant effect of the atmospheres on premiumness or on intent to purchase (Table 5, Figures 7E,F). These results failed to support hypotheses H1 and H2, in specialty coffee professionals.

Similar to Experiment 2, we also conducted a serial mediation analysis (Model 6 of Hayes, 2012; 10,000 bootstrap samples) (VR atmosphere → VR atmosphere fit → enjoyment of the experience → premiumness → intent to purchase). However, contrary to Experiment 2, the effects of VR city atmosphere (vs. control, indirect effect = 0.53, 95% CI [−0.53; 1.55] included zero), and VR farm atmosphere (vs. control, indirect effect = 1.03, 95% CI [−0.94; 2.60] included zero) on intent to purchase through VR atmosphere fit, enjoyment of the experience, and premiumness, as mediators were not significant. Those in the VR farm atmosphere reported significantly higher VR atmosphere fit than those in the control atmosphere (β = 1.83, *t* = 3.92, *p* <0.001). However, those in the VR city atmosphere did not report significantly higher VR atmosphere fit vs. the control (β = 0.95, *t* = 1.99, *p* = 0.06). Enjoyment of the experience was then regressed on VR atmosphere fit, but the direct effects were not significant (*p* = 0.16), as well as the direct effects of city (*p* = 0.20) and farm (*p* = 0.16) atmospheres. Neither VR atmosphere fit (*p* = 0.62), nor the enjoyment of the experience (*p* = 0.17) had any significant effects on premiumness. We only found direct effects of enjoyment of the experience (β = 0.39, *t* = 3.81, *p* <0.001) and premiumness (β = 14.36, *t* = 5.4, *p* <0.001) on intent to purchase.

#### 5.4.4. Sensory Evaluation

Contrary to Experiment 2, the analysis showed no significant effect of the virtual atmospheres in any of the sensory characteristics of the coffee (Table 5).

The results of Experiment 3 seemed to indicate that there is a higher fit between coffee and atmospheric cues that evoke its terroir, compared to the control but not to the city. The atmospheric cues did not influence intent to purchase in specialty coffee professionals. Experts seemed to enjoy the experience significantly more under the farm atmosphere vs. the control. However, there were no significant differences in the perception of premiumness or any of the sensory characteristics of the coffee.

## 6. General Discussion

In this study, we investigated the effect of visual atmospheric cues on the expectation and perception of premiumness, enjoyment of the experience, intent to purchase, and sensory evaluations of coffee in non-experts and specialty coffee professionals. We explored these effects using 2D online images and 360-degree VR atmospheres. We found that 2D visual cues evoking the broad origin of coffee (represented by a coffee farm) could influence premiumness expectations in non-expert consumers, supporting H1. Our results showed that VR atmospheres that portrayed the broad terroir of coffee, given by a farm (vs. a control atmosphere but not a city), could increase premiumness perception in non-experts—partially supporting H1—and the enjoyment of the experience in professionals—partially supporting H3. Moreover, for non-experts, both the farm and the city atmospheres indirectly increased the intent to purchase the coffee through coffee-VR atmosphere fit, enjoyment of the experience, and premiumness—partially supporting H2. Furthermore, these visual atmospheric cues influenced the sensory evaluation of coffee in non-experts in terms of sweetness and acidity.

In particular, we found that 2D visual cues that illustrated the broad origin of coffee presented high fit with the product and increased premiumness expectations. However, whether the image was from the specific origin of the coffee or not, and whether this image had a label indicating its location, had a negligible effect on fit and premiumness expectations. This being said, the relevant factor was that the images presented something that resembled the broad origin of the coffee (e.g., a coffee farm). Since non-experts may not be able to differentiate between images of urban or rural areas of countries that produce coffee, the location represented in the image did not influence premiumness expectations. Even with indications that the images were not from the specific origin of the coffee, other things equal, this discrepancy did not affect the fit and premiumness expectations.

Our results revealed differences among the VR atmospheres in terms of sense of presence. As one may expect at present, we found that atmospheres that portrayed the real world (i.e., the farm and the city), vs. computer-generated images (the control white room), triggered significantly higher sense of presence. These differences are not surprising since both the farm and the city atmospheres provided a richer experience, in terms of vividness, than the control atmosphere. More specifically, the difference in vividness is related to higher depth (Steuer, 1992), which is a key determinant for telepresence (Kim and Biocca, 1997; Willems et al., 2019). The control atmosphere consisted of a white, 3D computer-generated model of a small room. On the other hand, the other two atmospheres were high definition images of the real world with a wider variety of elements and details, as well as varied gamut of colors.

Telepresence is a crucial part of the online consumer experience (see Mollen and Wilson, 2010 for a review). Several studies have shown that telepresence can increase consumers' perception of product knowledge, attitudes, and intent to purchase (Klein, 2003; Suh and Chang, 2006; Animesh et al., 2011; Algharabat, 2018). Fiore et al. (2005) suggested that the sense of presence is related to the ability to provide sensory information about the product that might be particularly relevant for the evaluation of coffee in virtual environments.

### 6.1. Premiumness Associations

Following the concept of schema congruence, our a priori expectation was that the farm atmosphere was the most congruent with the coffee as they shared the common meaning of terroir or origin of the coffee (Mandler, 1982; Keller, 1993). Having said this, both the farm and the city atmospheres seemed to be somewhat congruent with the coffee as both presented significant differences compared to the control but not against each other. It is possible that consumers' schema related to coffee was not sufficient to share meaning with the farm atmosphere. Most people have never been to a coffee farm, and in effect, not sampled coffee in such a context. Moreover, people may have different expectations about how a coffee farm looks like. Furthermore, based on the concept of situational appropriateness (Giacalone and Jaeger, 2019b), it is possible that the city atmosphere was congruent with the coffee since coffee is more often consumed in city contexts.

Nevertheless, non-experts rated the coffee as more premium only under the farm atmosphere compared to the control. It is possible that despite participants' limited knowledge of coffee's terroir, some of them made associations between the coffee and something with nature that looked like a place where it may come from. As previous research has shown, indications of a product's origin can be associated with quality traits (Spielmann et al., 2014). Hence, they may have expected the coffee to be of high quality, which is a component of premiumness.

In the case of the control atmosphere, consumers might not have drawn any association with the coffee, which resulted in a lack of both (in)congruence and relevance. Studying the suitability between sponsors and events, Fleck and Quester (2007) suggested that congruence derives from two sources, namely expectancy and relevance. When unexpectedness is coupled with enough relevance to retain meaning, the resulting effect of moderate (vs. high or low) congruence is the most impactful (see also Gillespie et al., 2018; Zdravkovic et al., 2010). In our study, the lack of congruence and relevance from the control atmosphere could have led to the poorest evaluations of the coffee.

Another possible explanation can be derived from the curiosity hypothesis (Berlyne, 1966). The experience of the city atmosphere likely matched most consumers' expectations about coffee very closely, as it is a regular place for consumption, which may not have been remarkable and not have affected their hedonic state. However, the farm atmosphere may have been a surprise, or slight deviation from expectations, in the regular coffee experience, and could have resulted in increased curiosity and hence, a modestly higher degree of arousal (Schifferstein et al., 1999). More recently, as Hill et al. (2016) found, curiosity can generate more positive evaluations of products experiences and indirectly increase intent to purchase.

Contrary to the findings in non-experts, we did not find any effect of atmospheric cues in the perception of premiumness of coffee or its sensory evaluation in coffee professionals. It is likely that, given their specialized knowledge, coffee professionals examined more objective attributes of the coffee and could discriminate intrinsic factors relevant for the assessment of the coffee from irrelevant extrinsic cues (Lee and Lee, 2009). Furthermore, experts can use origin cues to weigh the quality of products for which origin is highly relevant, such as wine (D'Alessandro and Pecotich, 2013; Warman and Lewis, 2019). However, in our case, participants received no prior information regarding the coffee (e.g., origin, notes) or the atmospheres (e.g., location or whether they had some relationship with the coffee or not) that allowed them to make a product-origin match and make such assessments. Hence, it is likely they relied on an independent evaluation of the coffee without considering the atmospheres.

### 6.2. Enjoyment of the Experience

Product experiences are the sum of multiple factors, including the sensory elements of the products, associated emotions, and the meaning communicated to consumers (Hekkert and Schifferstein, 2008). The enjoyment of these experiences will be determined not just by the properties of the product itself, but by sensorial, emotional, cognitive, pragmatic, lifestyle, and relational dimensions (Gentile et al., 2007; De Keyser et al., 2015). On this line, Reinoso Carvalho et al. (2016) found that participants reported liking the sound-beer (congruent) tasting experience more when their attention was drawn toward both the beer and the music, as a single multisensory experience. This suggests that when people are asked to evaluate the experience as a whole, rather than the individual parts of it, congruence may influence enjoyment. Furthermore, meaning in the form of product/brand values and associations, as well as relationships and past experiences play a key role in product experiences. For instance, in a study exploring the drinking experience of beer, Gómez-Corona et al. (2017) found that the cognitive dimension is more salient for craft beers than for industrial ones and that consumers engage with the former through intellectual experiences and inquire about them (including their origin).

The context in which a product is consumed is another important factor for its experience. As Meiselman (2008) suggested, product experiences also relate to the interaction between a product and specific contexts and situations, which can influence the emotions people experience while consuming a product. More appropriate scenarios for food consumption can trigger more positive emotions (Torrico et al., 2020). Thus, the same product can be experienced differently depending on the context and situation.

Related to the present study, we did not find significant effects of the VR atmospheres on the enjoyment of the experience in non-experts. Consumers' knowledge of the value of the coffee's origin may be limited, so an atmosphere related to the terroir of coffee would not have much significance. Thus, consumers may have seen the coffee as a commodity product in isolation and not made a connection with the atmosphere to form an experience with rich meaning. Consumers may need to have a minimum degree of knowledge about the value of terroir in order to make sense of semiotic meanings of sense of place and frame consumption experiences (Smith Maguire, 2010; Charters et al., 2017). On the other hand, coffee professionals reported significantly higher ratings of enjoyment of the experience under the farm atmosphere compared to the control but not to the city. It is possible that the knowledge of coffee professionals about the value of terroir in coffee imbued the farm with greater context and meaning, so they did not see the coffee as a product in isolation but more as a holistic experience related to their specialized knowledge, which made it more enjoyable. These results are consistent with Wen and Leung (2021), who found consumers with higher levels of wine knowledge tend to appreciate virtual wine tours and the wine itself more than those consumers with lower levels of knowledge. The city could have also provided context and meaning to the experience, albeit a more routinely experience of drinking coffee in an everyday scenario that is not associated with the origin of the coffee and less so with specialty coffee.

### 6.3. From Visual Cues to Purchase Intent

The serial mediation results revealed that the city and farm atmospheres (vs. control) had significant positive effects on intent to purchase through VR atmosphere fit, enjoyment of the experience, and premiumness, in non-experts, partially supporting our three hypotheses. First, we found that VR atmosphere fit fully mediated the effect of the atmospheres on the enjoyment of the experience. Indeed, several studies have highlighted the importance of the characteristics of the contexts in which food is consumed for its enjoyment and evaluations (Meiselman et al., 2000; Edwards et al., 2003; Köster, 2003; Petit and Sieffermann, 2007; Piqueras-Fiszman and Jaeger, 2014a,b). For instance, Piqueras-Fiszman and Jaeger (2014a) suggested that higher perceived appropriateness between a product and the context in which it is consumed can elicit more positive emotions. Our results with non-experts also confirm the role of fit between product and place of origin on product's evaluations (Häubl and Elrod, 1999; Chen and Tsai, 2007; Johnson et al., 2016; Spielmann, 2016) and indicate that the enjoyment of the experience is positively related to these evaluations.

In the case of coffee professionals, the effect of the atmospheres was not mediated by the fit between the coffee and the VR atmosphere, which as mentioned before, confirms the importance of the meaning of the atmospheres themselves coupled with previous knowledge. Furthermore, we found that VR atmosphere fit, as well as enjoyment of the experience mediated the effect of the atmospheres on the expectation of premiumness. These results are in line with previous studies showing the positive impact of fit and enjoyment of the experience on premiumness perception (Creusen et al., 2018). Finally, we observed direct effects of premiumness and enjoyment of the experience on intent to purchase.

Overall, it is likely that the congruence between the coffee and the atmospheres reduced the cognitive effort required to make sense of the premium experience, leading to a higher enjoyment and subsequently to better product evaluations that in the end increased intent to purchase. These results bring support to previous research that highlighted the interest of using images about a specific location to nudge toward higher intent to purchase (Hosany et al., 2006; Chen and Tsai, 2007). Moreover, they are consistent with previous studies that have shown the essential role of marketing experiences on intent to purchase (Schmitt, 1999; Babin and Attaway, 2000; Turley and Milliman, 2000), in particular in the luxury sector, where experiences play an important role in the perceived quality of the product (Vigneron and Johnson, 2004; Christodoulides et al., 2009; Hung et al., 2011). As Lee et al. (2015) suggested, consumers derive value from luxury goods from hedonic and symbolic attributes, besides quality benefits. Moreover, cues about the origin of the product convey important information in luxury goods (Godey et al., 2012). The perceived level of fit between a brand and its origin will influence affective behaviors toward the brand and subsequently intent to purchase (Siew et al., 2018).

### 6.4. Sensory Evaluation

While taste refers to what happens in the tongue, consumers can attribute taste qualities to odors mainly because odors are associated with tastes when they are experienced in tandem by eating (Stevenson et al., 1999). Moreover, consumers use attributes or descriptors related to other senses to describe olfactory stimuli due to the multiple crossmodal correspondences between the senses (Velasco et al., 2014; see also Deroy et al., 2013). This relationship between taste and smell allows consumers to form tastes expectations of food and beverage products by smelling them. In our study with non-experts, we obtained unexpected findings regarding the sensory evaluation of coffee aromas. Participants found the coffee more acidic under the farm atmosphere compared to the control. Moreover, they evaluated the coffee under the city atmosphere as significantly less sweet compared to the control. The results in the evaluation of sweetness may have arisen due to the varying degrees of sensory complexity of the different atmospheres.

In the farm atmosphere, it is possible that a sensation transference effect was at play and predominant colors in the atmospheres influenced participants' tastes perception (Cheskin, 1957; Chen et al., 2018). Indeed, Chen et al. (2020) found that sweet-congruent (i.e., round shapes and red colors) VR environments can increase the perception of sweetness of beverages (i.e., grenadine juice). In our case, it is possible that participants associated the high amount of green color with expectations of acidity of the coffee. Research suggests that there is a color-basic taste crossmodal correspondence between green and sourness/acidity (Spence et al., 2015; Saluja and Stevenson, 2018) that can influence drinking experiences (Spence et al., 2014; Carvalho and Spence, 2019).

A potential explanation for the lower sweetness ratings in the city atmosphere is that the latter, although only visual, evoked the perception of loud background noise or perhaps sensory overload, thus reducing the perception of sweetness. Several studies have highlighted that visual cues can facilitate mental simulation (Elder and Krishna, 2012; Cian et al., 2014; Xie et al., 2016; Petit et al., 2017, 2018; Palcu et al., 2019). In the same way, 3D images (VR and AR) appear to stimulate mental simulation (Choi and Taylor, 2014; Heller et al., 2019). Furthermore, as previous literature suggests, loud noises can affect the perception of sweetness (Woods et al., 2011; Stafford et al., 2013; Velasco et al., 2014; Yan and Dando, 2015; Lin et al., 2019; Xu et al., 2019).

## 7. Limitations and Research Perspectives

One of the main limitations of the present study relates to the atmospheres used in Experiments 2 and 3. The response of participants, as well as the fit of the VR atmospheres with coffee, was likely to be dependent on the specific stimuli presented. For instance, some people might have had a special connection to a specific city presented, and others might have had special memories from farms. Expanding this study to explore atmospheres completely unrelated or negatively associated with coffee could yield interesting results. Moreover, while we explored different levels of fit with the stimuli, there was no control condition in Experiment 1. Exploring the effects of stimuli unrelated, or with no context, with respect to the coffee would provide a better control for the effect of origin cues.

It should also be noted that in Experiments 2 and 3, we only tested one coffee sample. Additionally, in Experiment 2, the task consisted of smelling the coffee, whereas the task in Experiment 3 was to taste the coffee. This provides consistent insights associated with different stages of the specialty coffee journey. However, to ensure that results are not coffee and origin dependent, future studies should involve coffees from different places of origin and incorporate smelling and tasting for both the non-experts and professionals.

Furthermore, different elements in the atmospheres could not be fully controlled. For example, the atmospheres diverged in lighting, colors, number of people, and proximity to these people, among other factors. Other elements not controlled for in the atmospheres were the overall hue and brightness of our different atmospheres, which could have affected participants' perception of product premiumness and intent to purchase, as well as experience enjoyment (cf. Spence et al., 2014). In our study, the coffee could have been evaluated better under the city atmosphere due to the colorfulness and increased color lightness in the city atmosphere compared to the farm. Several studies have highlighted the effect of colors on consumers' expectations and perceptions of packaged products (Gatti et al., 2014; Tijssen et al., 2017). For example, Paakki et al. (2019) suggested that more colorful products are more attractive and are preferred to less colorful ones. Motoki et al. (2019) also suggested that consumers prefer light-colored products compared to dark-colored ones. Future studies should be conducted to control for how the overall color space in the atmosphere can affect premiumness perception.

Despite the support of a prominent specialty green coffee sourcing company, recruiting coffee professionals was challenging, which resulted in a low sample size in Experiment 3. Nevertheless, our results serve as an initial step to study the effect of virtual atmospheric cues on the perception of premiumness, enjoyment of product experiences, and intent to purchase. Future studies can further explore differences between non-experts and professionals through studies with more statistical power. Extending this research to products for which terroir is crucial vs. other products for which it is less important could also provide exciting results. Moreover, further studies could explore whether highlighting the maker of a product has a greater impact than highlighting the place of production in manufactured products. Exploring the possibilities of using multisensory enabling technologies with other senses, such as touch, could also generate relevant insights (Petit et al., 2019).

## 8. Implications for Industry

The use of VR is still in its early stages in many industries, but its relevance and degree of implementation is growing (Boyd and Koles, 2019). Hence, it is imperative that practitioners are aware of key factors that make effective virtual experiences. Our findings are highly relevant and lead to interesting reflections for industry as they can help guide the creation of product-based VR experiences. Developers may aim to enhance the sense of presence of virtual experiences since it can influence their quality and enjoyment. As our results suggested, physical realism and consistency with the real world are critical aspects in generating a high sense of presence in these experiences. While VR can be used to develop non-worlds, developers and marketers should strive to create relevant connections between virtual experiences and their underlying products in terms shared meaning and appropriate contexts.

While our results did not reveal a significant difference between the farm and the city atmospheres, inexpensive versions of VR can be used to attract and introduce new consumers to specialty coffee and communicate the added value of terroir by engaging them in novel immersive experiences. These inexpensive solutions can make the coffee experience at home more enjoyable by portraying relevant scenarios, whether they are related to the origin of coffee or to consumption contexts.

## Data Availability Statement

The datasets generated and analyzed in this study can be found in OSF at https://osf.io/5vne6/.

## Ethics Statement

The studies involving human participants were reviewed and approved by Ethics Committee of BI Norwegian Business School. The patients provided their written informed consent to participate in this study. Written informed consent was obtained from the individual(s) for the publication of any potentially identifiable images or data included in this article.

## Author Contributions

FBE: conceptualization, methodology, formal analysis, investigation, writing-original draft, writing-review and editing, and visualization. OP: methodology, validation, formal analysis, resources, writing-review and editing, and visualization. CV: conceptualization, methodology, validation, formal analysis, investigation, resources, writing-review and editing, visualization, supervision, and funding acquisition. All authors contributed to the article and approved the submitted version.

## Conflict of Interest

The authors declare that the research was conducted in the absence of any commercial or financial relationships that could be construed as a potential conflict of interest.

## Footnotes

^1^The required sample size for Experiments 1 and 2 were determined using GPower 3.1.9 (Faul et al., 2007) using an alpha level of 0.05 and 0.95 (1 - β) statistical power. The analysis indicated sample sizes of 640 and 150, for Experiments 1 and 2, respectively, were required. Moreover, recent research on sensory and consumer science studies (Velasco et al., 2018; Motoki et al., 2020b) use similar sample sizes.

^2^Extracted at second 46 of the 360-degree video “Come On In—Meet Coffee Farmer Samuel—360°” from the Fairtrade Association (https://youtu.be/LCmU-uK93wc?t=46).

^3^Extracted at second 30 from the video “EXTRA 5K 360 VR Video Times Square Manhattan New York Downtown Manhattan 2018 USA NYC 4k Jonnysbazar” (https://youtu.be/-UY0pzJdK7Y?t=30).

^4^Extracted from the video “White Room” (https://youtu.be/e6xduAUJUyg).
