# Supplementary material for: Virtual Terroir and the Premium Coffee Experience
Source: Front Psychol. 2021 Mar 18;12:586983. doi: 10.3389/fpsyg.2021.586983 (PMC8013734; doi:10.3389/fpsyg.2021.586983)
Supplement: Supplementary file 1 [file Data_Sheet_1.PDF]

Supplementary Material

1 MAIN TASK IN EXPERIMENT 1

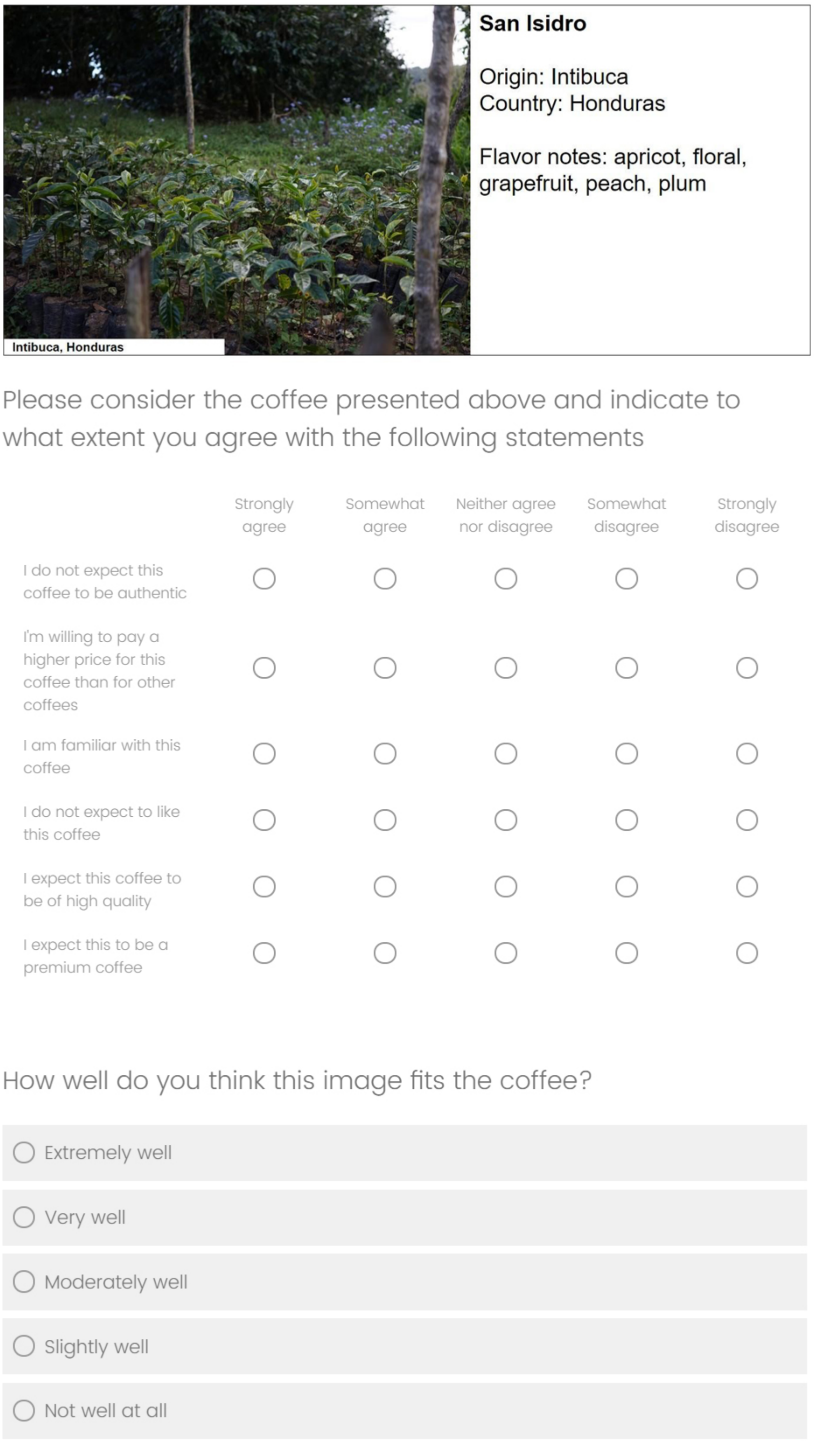

Figure S1. Snapshot of main task in Experiment 1 consisting of premiumness and fit evaluations

2 PARTICIPANT'S PROFILE AND RELATIONSHIP WITH COFFEE IN EXPERIMENT 1

In order to get a sense of the profile of respondents and their relationship with specialty coffee, we included questions regarding their coffee consumption habits and motivations using a five-point Likert scale. First, respondents were asked to indicate how often they consumed coffee by selecting the most appropriate option from "Never" to "2 or more times a day". Then, participants indicated their motivations for drinking coffee (to wake up, for pleasure, on special occasions, by habit). Additionally, participants ranked the factors they considered most important when purchasing coffee (brand, coffee format, origin of the coffee, packaging, price, production type, quality, taste). They also indicated how familiar they were with specialty coffee and with each of the four countries of origin of the coffees presented (Burundi, El Salvador, Honduras, Kenya) from "Not familiar at all" to "Extremely familiar".

An analysis of the participants' characteristics revealed that there were no major differences among the groups in terms of coffee consumption behaviours and motivations (S1). Overall, participants were moderate to strong coffee consumers ($M = 5.14, SD = 2.15$). They appeared to drink coffee for pleasure ($M = 3.79, SD = 1.37$) and reported liking the taste of coffee ($M = 4.07, SD = 1.35$). However, participants presented low familiarity with specialty coffee ($M = 2.32, SD = 1.06$) and moderate consumption of premium coffee ($M = 3.09, SD = 1.29$).There was an indication that participants drank coffee to wake up ($M = 3.19, SD = 1.55$) and that they considered drinking coffee as a habit ($M = 2.97, SD = 1.52$). Furthermore, participants indicated that the most important factors they consider when purchasing coffee were taste, quality, and price.

Table S1. Participants' coffee consumption habits and motivations in Experiment 1. Elements sharing a letter are not significantly different

	No Origin				Origin			
	City		Farm		City		Farm	
	Label	No label	Label	No label	Label	No label	Label	No label
Frequency of Coffee Consumption	4.91^{a}	5.35^{ab}	5.14^{ab}	5.19^{ab}	4.96^{ab}	5.27^{ab}	5.37^{b}	4.95^{ab}
Familiar with specialty coffee	2.19^{a}	2.41^{ab}	2.39^{ab}	2.45^{b}	2.18^{a}	2.35^{ab}	2.3^{ab}	2.33^{ab}
Likes the taste of coffee	4.06^{a}	4.1^{a}	4.11^{a}	4.02^{a}	3.92^{a}	4.03^{a}	4.12^{a}	4.19^{a}
Consumes Premium Coffee	2.99^{abc}	3.26^{c}	3.1^{abc}	3.12^{abc}	2.97^{ab}	3.24^{bc}	3.08^{abc}	2.94^{a}
Drinks coffee for pleasure	3.82^{b}	3.8^{ab}	3.84^{b}	3.85^{b}	3.51^{a}	3.89^{b}	3.82^{b}	3.77^{ab}
Drinks coffee in special occasions	2.9^{bc}	2.66^{ab}	2.98^{c}	2.99^{c}	2.57^{a}	2.95^{c}	2.87^{bc}	2.85^{abc}
Drinks coffee as a habit	2.95^{a}	2.95^{a}	2.98^{a}	3.15^{a}	2.85^{a}	2.99^{a}	3.07^{a}	2.86^{a}
Drinks coffee to wake up	3.06^{a}	3.07^{ab}	3.21^{ab}	3.32^{ab}	3.13^{ab}	3.29^{ab}	3.39^{b}	3.04^{a}

3 EFFECTS OF INDEPENDENT COMPONENTS OF PREMIUMNESS INDEX IN EXPERIMENT 2

Table S2. Results of independent ANOVAs on the different components of the premiumness index and linking

	F	p	η_p^2
Quality	**4.60**	**0.012**	**0.062**
Authenticity	2.29	0.105	0.032
Premiumness	**3.43**	**0.035**	**0.047**
Willingness to pay a premium	2.06	0.132	0.029
Liking	0.73	0.485	0.010
