# Supplementary figures and images for: Virtual Terroir and the Premium Coffee Experience
Source: Front Psychol. 2021 Mar 18;12:586983. doi: 10.3389/fpsyg.2021.586983 (PMC8013734; doi:10.3389/fpsyg.2021.586983)

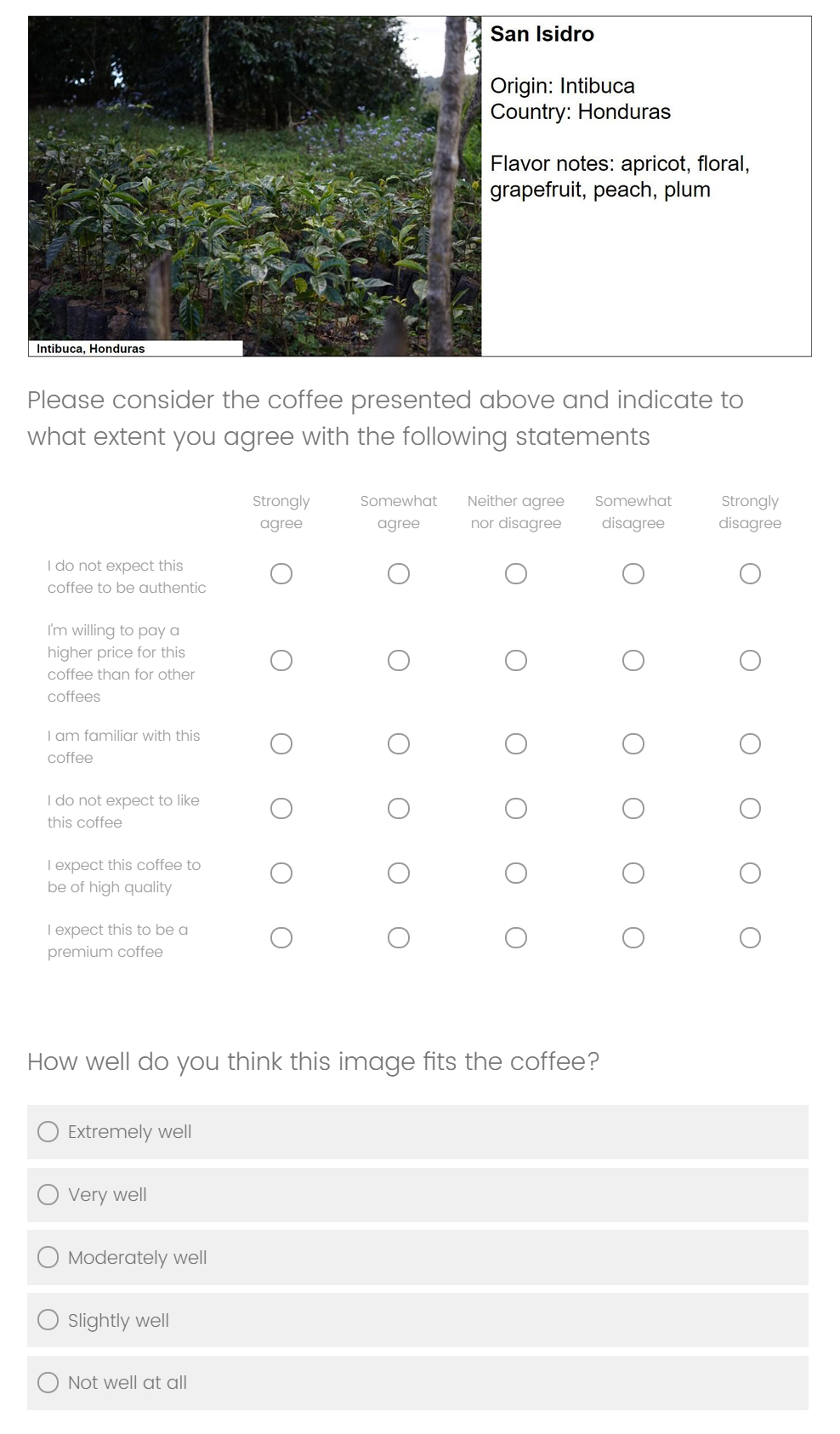

Supplement: Supplementary file 2 [file Image_1.JPEG]
